# Myxosortase: an intramembrane protease that sorts MYXO-CTERM proteins to the cell surface

**DOI:** 10.1128/mbio.04067-24

**Published:** 2025-03-12

**Authors:** Tingting Guo, Daniel H. Haft, Daniel Wall

**Affiliations:** 1 Department of Molecular Biology, University of Wyoming, Laramie, Wyoming, USA; 2 National Center for Biotechnology Information, National Library of Medicine, National Institutes of Health, Bethesda, Maryland, USA; University of Georgia, Athens, Georgia, USA

**Keywords:** *Myxococcus xanthus*, myxosortase, cell surface protein, outer membrane exchange, protein sorting, sortase

## Abstract

Cell surface proteins determine how cells interact with their biotic and abiotic environments. In social myxobacteria, a C-terminal protein sorting tag called MYXO-CTERM is universally found within the Myxococcota phylum, where their genomes typically contain dozens of proteins with this motif. MYXO-CTERM harbors a tripartite architecture: a short signature motif containing an invariant cysteine, followed by a transmembrane helix and a short arginine-rich C-terminal region localized in the cytoplasm. In *Myxococcus xanthus*, MYXO-CTERM is predicted to be posttranslationally lipidated and cleaved for subsequent cell surface localization by the type II secretion system. Here, following our bioinformatic discovery, we experimentally show that myxosortase (MrtX, MXAN_2755) is responsible for the C-terminal cleavage and cell surface anchoring of TraA, a prototypic cell surface receptor. The cleavage by MrtX depends on conserved cysteines within the MYXO-CTERM motif of TraA. *M. xanthus* mutants lacking myxosortase are defective in TraA-mediated outer membrane exchange and exhibit cell envelope defects. In a heterologous *Escherichia coli* expression system, the MYXO-CTERM motif is cleaved when MrtX is co-expressed. Therefore, MrtX represents a new family of sorting enzyme that enables cell surface localization of MYXO-CTERM proteins.

**IMPORTANCE:**

The CPBP (CaaX protease and bacteriocin processing) protease family is widespread across the three domains of life. Despite considerable research on eukaryotic homologs, prokaryotic CPBP family members remain largely unexplored. In this study, we experimentally reveal the function of a novel CPBP protease called myxosortase. Our findings show that myxosortase is responsible for the C-terminal cleavage and cell surface anchoring of substrate proteins containing MYXO-CTERM motifs in *Myxococcus xanthus*. MYXO-CTERM cleavage also occurred in a heterologous *Escherichia coli* host when myxosortase is co-expressed. This is the first report that a CPBP protease is involved in protein sorting in prokaryotes. This work provides important insights into the biogenesis and anchoring of cell surface proteins in gram-negative bacteria.

## INTRODUCTION

Cell surface proteins allow cells to recognize and interact with their surrounding environments. Proper protein surface localization is critical for many biological processes, including motility, adhesion, pathogenesis, nutrient uptake, kin recognition, and intercellular communication. Transport and anchoring of these proteins to the cell surface occur by various mechanisms (1, 2). One common mechanism, found in eukaryotes and prokaryotes, depends on posttranslationally modified C-terminal sorting tags that anchor protein to the cell surface (3–5).

In prokaryotes, sortase, rhombosortase, and the exosortase/archaeosortase families are three unrelated types of membrane-bound proteases that process C-terminally tagged proteins and mediate their cell surface attachment. In *Staphylococcus aureus*, the cysteine endopeptidase Sortase A (EC 3.4.22.70) was the first to be identified and serves as a prototypic sorting enzyme. Sortase A has extracytoplasmic transpeptidase activity that cleaves and covalently attaches proteins to the cell wall. Substrate proteins typically contain an N-terminal signal peptide for Sec-dependent secretion across the cytoplasmic membrane and a conserved C-terminal tripartite architecture, consisting of a signature LPXTG motif, a hydrophobic transmembrane helix, and a cluster of cytoplasmic basic residues (6–8). This sortase system is widespread among gram-positive bacteria (9) and thus provides a common mechanism for displaying proteins on the cell surface. In gram-negative bacteria, exosortases were identified through comparative genomic studies as multiple membrane-spanning cysteine endopeptidases that link the tripartite C-terminal protein-sorting signal PEP-CTERM to the production of exopolysaccharide (10, 11). Archaeosortases are exosortase-like enzymes found in archaea (12). In *Haloferax volcanii*, the archaeosortase ArtA was demonstrated to recognize the PGF-CTERM sorting signal, with its Pro-Gly-Phe signature motif, followed by a hydrophobic region and then positively charged residues (12–14). In *Vibrio cholerae*, the intramembrane serine protease rhombosortase RssP (EC 3.4.21) recognizes the GlyGly-CTERM sorting signal, which also exhibits tripartite architecture (15). Exosortases, archaeosortases, and rhombosortases all appear to leave their substrates on the cell surface, anchored into the membrane, suggesting each may function as a transpeptidase, as sortase does, leaving some new moiety attached at the C-terminal region cleavage site (16).

**Fig 1:**
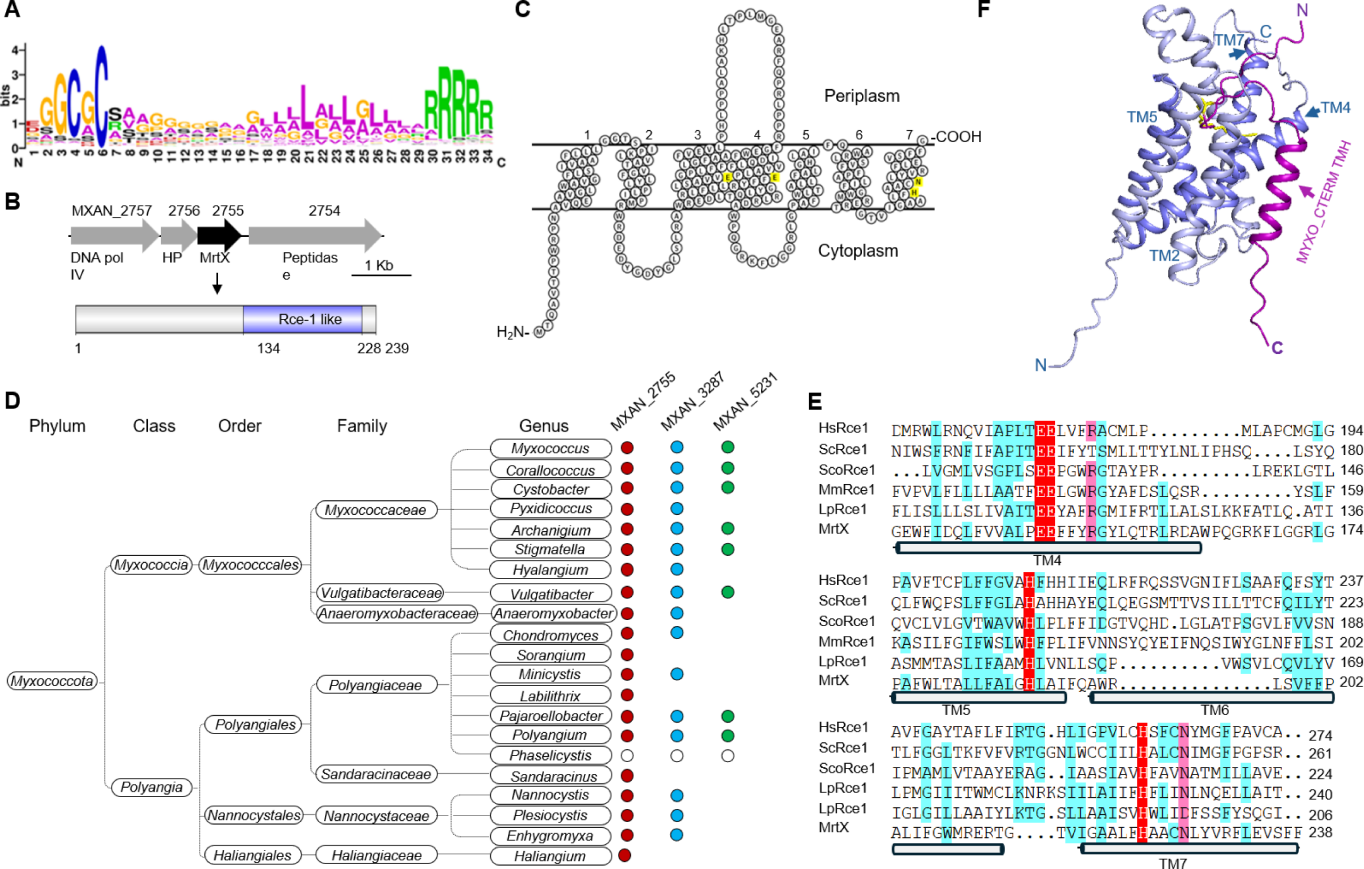
MrtX structure and characterization. (**A**) MYXO-CTERM sequence logo from reference 16. (**B**) Genetic organization of the *mrtX* locus in *M. xanthus*. (**C**) MrtX membrane topology based on AlphaFold 3 predictions and drawn with Protter (22). Putative transmembrane segments are numbered. Four invariant residues among CAAX family members highlighted in yellow. (**D**) Distribution of CAAX genes across the phylum Myxococcota (23). The presence of MXAN_2755 (*mrtX*) orthologs (red; all <10^−17^ E-values), MXAN_3287, and MXAN_5231 (cutoff value < 10^−5^) are indicated in blue and green, respectively. Blank, not known; genome unavailable. (**E**) Multiple sequence alignment of the CPBP domains from Rce1 homologs representing all three domains of life: *Homo sapiens* RCE1 (HsRce1; UniProt ID Q9Y256), *Streptomyces coelicolor* Rce1 (ScoRce1; UniProt ID Q9XAK4), *Saccharomyces cerevisiae* Rce1p (ScRce1; UniProt ID Q03530), *Methanococcus maripaludis* Rce1 (MmRce1; UniProt ID Q6LZY8), *Lactobacillus plantarum* Rce1 (LpRce1; UniProt ID C6VK86), and *M. xanthus* MrtX (UniProt ID A0A7Y4IEC4). Invariant catalytic residues are highlighted in red, and the conserved residues are shaded purple (homology level > 75%) and blue (homology level > 50%). (**F**) AlphaFold 3 co-structure of MrtX (blue) with the MYXO-CTERM peptide of TraA GTEPQGGAFC(palmitoyl; yellow)CGTTADATPAGSTTFLLLLAAGLTFLRPRRPAR (magenta). TM4, TM5, TM6, and TM7 constitute the CPBP domain.

*Myxococcus xanthus* is a gram-negative soil bacterium renowned for its complex social behaviors, including microbial predation, aggregative multicellular development, and kin recognition. To form social groups of clonal cells and discriminate against nonkin, they use a polymorphic cell surface protein called TraA and its cohort protein TraB (17). Specifically, TraA serves as a kin recognition receptor by homotypic binding to neighboring cells that present an identical or compatible allele of TraA (18, 19). Following TraA-TraA recognition, cells undergo outer membrane exchange (OME), a process whereby OM proteins and lipids are bidirectionally transferred between compatible or clonal cells (19). OME requires that TraA is correctly processed and localized on the cell surface. The MYXO-CTERM motif is a myxobacteria-specific protein sorting tag that directs TraA and other proteins with this motif to the cell surface (Fig. 1A) (20). Notably, when expressed without its MYXO-CTERM motif, TraA is released to the extracellular milieu mediated by the type II secretion system (T2SS), abolishing cell surface localization and function (20). Similar to the mentioned sorting tags, MYXO-CTERM (https://www.ncbi.nlm.nih.gov/protfam/?term=TIGR03901.2) has a consensus tripartite structure (Fig. 1A). This sequence comprises a short signature motif, containing an invariant cysteine, followed by a hydrophobic transmembrane helix and an arginine-rich cytoplasmic cluster (21).

Previous experimental work had not identified the MYXO-CTERM processing enzyme. However, our recent *in silico* study predicted that the MYXO-CTERM is cleaved by myxosortase, MrtX (MXAN_2755), presumably not in a sortase-like one-step transpeptidation, but instead following lipidation of the invariant Cys by an unknown enzyme (16). In that study, we showed that a conserved two-gene neighborhood, consisting of just MrtJ and a single JDVT-CTERM protein (a sorting enzyme and a target protein), occurs sporadically in both beta- and gamma-proteobacteria, although never in the Myxococcota. MrtJ and MrtX are closely related members of the CPBP (CAAX protease and bacteriocin-processing) family of glutamic acid intramembrane proteases, which also includes the eukaryotic CAAX prenyl protease Rce1. Partial phylogenetic profiling (PPP) (10), a data mining method, identified MrtX in *M. xanthus* and MrtC in *Chondromyces crocatus* as the only probable intramembrane proteases in the small sets of proteins with their best matches exclusively in other species with MYXO-CTERM proteins. Because of the strong sequence conservation among MrtJ and the C-terminal regions of MrtX and MrtC, as well as the invariant Cys residue shared by JDVT-CTERM and MYXO-CTERM, we hypothesized that MrtX is the myxosortase of *M. xanthu*s (16), and here we sought to experimentally test this prediction.

The laboratory *M. xanthus* strain contains 35 proteins, including TraA, with MYXO-CTERM motifs, all of which contain N-terminal signal peptides (Table S1). Most of these proteins are predicted to function as cell surface adhesins or hydrolases involved in diverse social interactions. For example, MepA is an extracellular protease implicated in predation (24). MstC is involved in cell-cell cohesion, social (S) motility, which is powered by type IV pili retraction, and development (25). Although these and other MYXO-CTERM proteins are likely involved in important cell behaviors, knowledge gaps remain in understanding the mechanism of sorting and anchoring them to the cell surface.

Here, we experimentally show that myxosortase is responsible for processing MYXO-CTERM-containing proteins. Our findings show that mutations in *mrtX* inhibit TraA processing, localization, and function. *mrtX* mutants also exhibit cell envelope defects. Finally, MrtX expression in *Escherichia coli* results in cleavage of MYXO-CTERM substrates near the C-terminus, which collectively shows that MrtX processes MYXO-CTERM proteins.

## RESULTS

### MrtX (myxosortase) plays a role in TraA-mediated OME

The *mrtX* gene (MXAN_2755; NCBIfam NF040593; https://www.ncbi.nlm.nih.gov/protfam/?term=NF040593) encodes a transmembrane protein of 239 amino acids with a C-terminal CAAX (Cys-aliphatic-aliphatic-any) protease domain (Rce1-like) with four putative catalytic residues located within its transmembrane helixes (Fig. 1B and C) (16). Orthologs of *mrtX* are found in all known genera within the Myxococcota phylum (Fig. 1D). In the *Myxococcus*, *Corallococcu*s, and *Pyxidicoccus* genomes, *mrtX* was typically located within a cluster of three genes (Fig. 1B). MrtX is homologous and structurally similar to MmRce1 (16), a member of the glutamic endopeptidase family (EC 3.4.26-) that also includes the eukaryotic type II CAAX protease Rce1 (EC 3.4.26.1). To illustrate conserved residues, sequence alignments of the CPBP domain from representative proteases across the three domains of life were selected. Their alignment (Fig. 1E) shows that E146, E147, H223, and N227 belong to two well-conserved motifs, previously described in an extensive comparison of type II CAAX prenyl protease homologs (26) and later shown to be critical for catalytic activity, as seen from the structure and characterization of *Methanococcus maripaludis* MmRce1 (27). These residues are key to understanding and defining a new category of proteases, the intramembrane glutamic endopeptidases. Membrane topology predictions indicate that these conserved residues reside in the membrane (Fig. 1C), consistent with them being intramembrane proteases. Similar to MmRce1, the EExxxR and HxxxN motifs of MrtX were located within TM4 and TM7, respectively (Fig. 1C and E).

To understand how MrtX recognizes substrates, we used AlphaFold 3 (28) to model it with a peptide based on the MYXO-CTERM sequence of TraA , where a palmitoyl lipid was attached to the invariant cysteine residue. The resulting model aligns with the proposed catalytic mechanism of the CPBP protein (27) and suggests that the palmitoyl lipid, or similar lipid, enters the MrtX catalytic site from the membrane, sealing the gap between the nonpolar surfaces of TM2 and TM4 (Fig. 1F). Structural superposition of MrtX and MmRce1 shows that their overall architectures were similar (16), with all invariant residues well aligned to form the catalytic pocket (Fig. S1A). Similarly, an oxyanion hole was formed by H223 and N227 within its invariant HX3N motif, located on TM7 (Fig. S1A and B). Similar to MmRce1, E146 and H188 bridge a water molecule directly (Fig. S1B) (27), forming an acid-base-nucleophilic triad to hydrolyze the MYXO-CTERM peptide.

Myxobacteria genomes typically contain dozens of MYXO-CTERM proteins and up to 73 in the case of *C. crocatus* (16). In contrast, myxobacteria with unusually reduced genome sizes and limited social behaviors contain few MYXO-CTERM proteins, e.g., three to six. These examples include *Anaeromyxobacter* spp., where, notably, adjacent ORFs to *mrtX* contain MYXO-CTERM motifs that were divergently transcribed. In rare cases, ORFs contain internal MYXO-CTERM motifs (discussed below).

**Fig 2:**
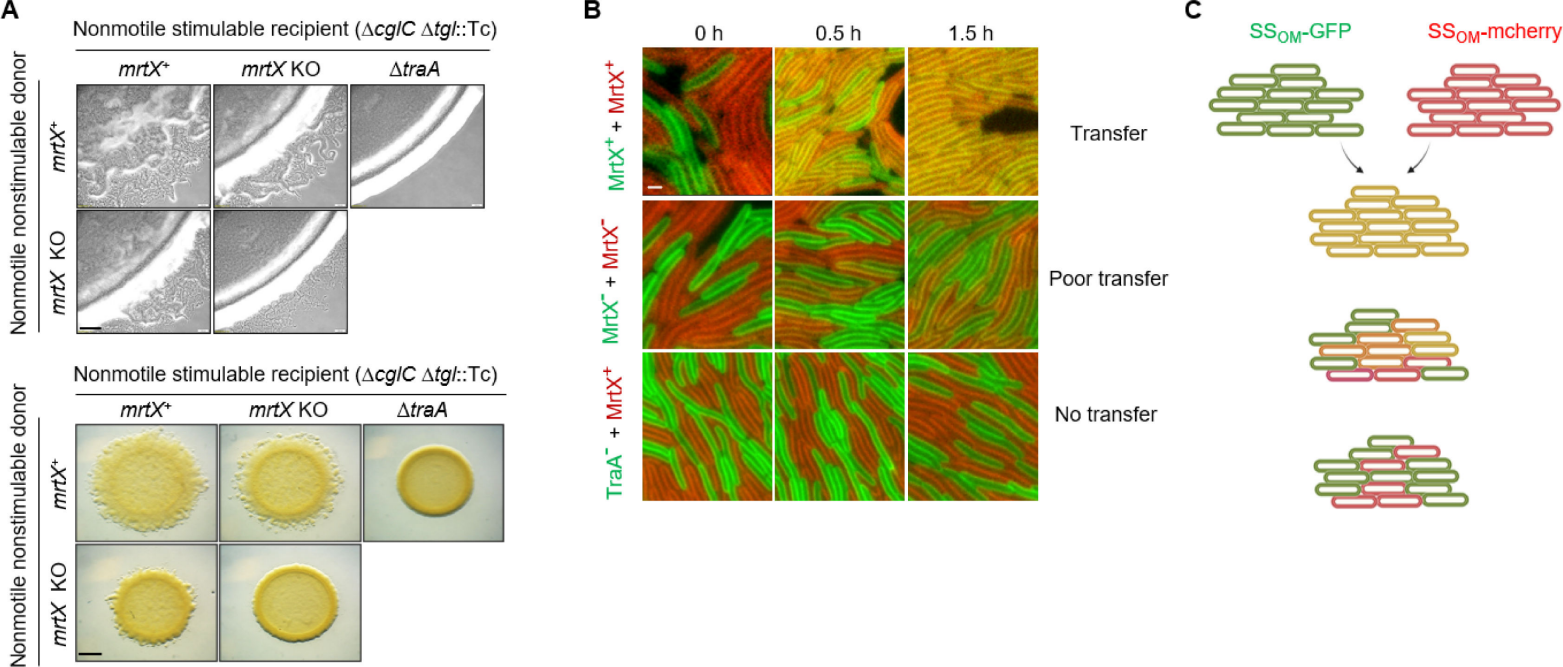
MrtX function in TraA-mediated OME. (**A**) Stimulation assays to assess *mrtX* role in TraA-mediated OME. Positive control, donor, and recipients competent for OME (emergent cell flares from inoculum); negative control donor lacks *traA* (sharp colony edge). *mrtX* genotype shown for donor and recipient strains. Recipient contains two mutations (Δ*cglC* Δ*tgl*) that abolish A- and S-motility that are restored by OME from nonmotile donors that contain the cognate WT *cglC* and *tgl* alleles. See Table S2 for strain details. Scale bars, 200 µm and 0.5 mm, respectively. (**B**) OME between *mrtX*^+^ cells (top) and *mrtX*^−^ cells (middle). The Δ*traA* control strain abolishes OME (bottom). Strains were labeled with either SS_OM_-mCherry or SS_OM_-GFP; OM lipoprotein reporters for OME. Transfer was assessed by the ability of cells to obtain both reporters and hence turn yellow. Merged images of green and red channels. See Fig. S3 for single channel images. Scale bar, 2 µm. (**C**) Schematic of OME at 1.5 h illustrating three outcomes.

We sought to experimentally test our *in silico* prediction that myxosortase cleaves MYXO-CTERM motifs. To do so, we used TraA as a model substrate. We constructed *mrtX* insertion mutants and tested for TraA-dependent OME function in stimulation assays (21). Here, we mixed a nonmotile, nonstimulatable donor with a nonmotile recipient mutant that can be transiently stimulated for adventurous (A) gliding motility and S-motility (29). Stimulation, or extracellular complementation, occurs where OME transfers missing motility proteins in the recipient (Δ*cglC* Δ*tgl*) from donor cells that contain the corresponding wild-type (WT) CglC and Tgl lipoproteins. As mentioned, OME requires functional TraA proteins in both donor and recipient cells. Thus, OME defects resulted in reduced or blocked stimulation as judged by the inability of recipient cells to swarm out from the mixed colony edge on agar. Notably, compared to the parent control, when either the donor or recipient strain contains an *mrtX* mutation, stimulation was impaired, particularly when donor cells contain the *mrtX* mutation (Fig. 2A). In contrast, when both donor and recipient strains contain *mrtX* mutations, stimulation was severely defective (Fig. 2A), though not abolished.

To better quantify the phenotype, the donor strains were mixed with a recipient strain at different ratios. At both 24 and 72 h, as the ratio of donor cells decreased, stimulation correspondingly decreased. Compared to the *mrtX*^+^ control, stimulation was decreased when the donor strain contained an *mrtX* mutation. As judged in this assay, the *mrtX* mutant reduced stimulation by ~10-fold compared to its parent control (Fig. S2).

In a second assay for TraA-mediated OME, we labeled two populations with SS_OM_-mCherry or SS_OM_-GFP lipoprotein reporters that are readily transferable between cells by OME (30). Initially, following mixing and plating, these populations were distinct (Fig. 2B and C; Fig. S3). However, after 90 minutes, the majority of *mrtX* cells remained visibly distinct, indicating a defect in OME. This finding contrasts with the WT positive control where the two populations were indistinguishable and phenotypically uniform resulting from bidirectional OME of reporters. As a negative control, one strain contained a *traA* mutation and consequently, no OME occurred. Furthermore, *mrtX* mutants displayed no significant difference in double-labeled cells over time, unlike the positive control (Fig. S3). Taken together, these results show that *mtrX* is critical for efficient OME, which is mediated by TraA in *M. xanthus*.

The above results showed residual OME activity in *mrtX* mutants, suggesting some TraA cell surface localization and activity remained. The *M. xanthus* genome contains two other CPBP family intramembrane glutamic endoproteases, MXAN_3287 (WP_011553332.1) and MXAN_5231 (WP_011555198.1) (16). Although these proteins share the CAAX protease-like CPBP domain, they have low sequence similarity to MrtX and are not universally conserved across the Myxococcota phylum like MrtX (Fig. 1D) (16). Nevertheless, since Δ*mrtX* mutants showed a partial stimulation defect, we hypothesized that another CAAX endopeptidase might process MYXO-CTERM. To test this, we constructed insertion mutants in MXAN_3287 or MXAN_5231 for both the donor and recipient strains in Δ*mrtX* or *mrtX*^+^ backgrounds. However, no additive defect was observed (Fig. S4), indicating that neither proteins affect TraA function. Thus, MrtX is the primary myxosortase for TraA MYXO-CTERM processing.

### MrtX cleaves TraA

**Fig 3:**
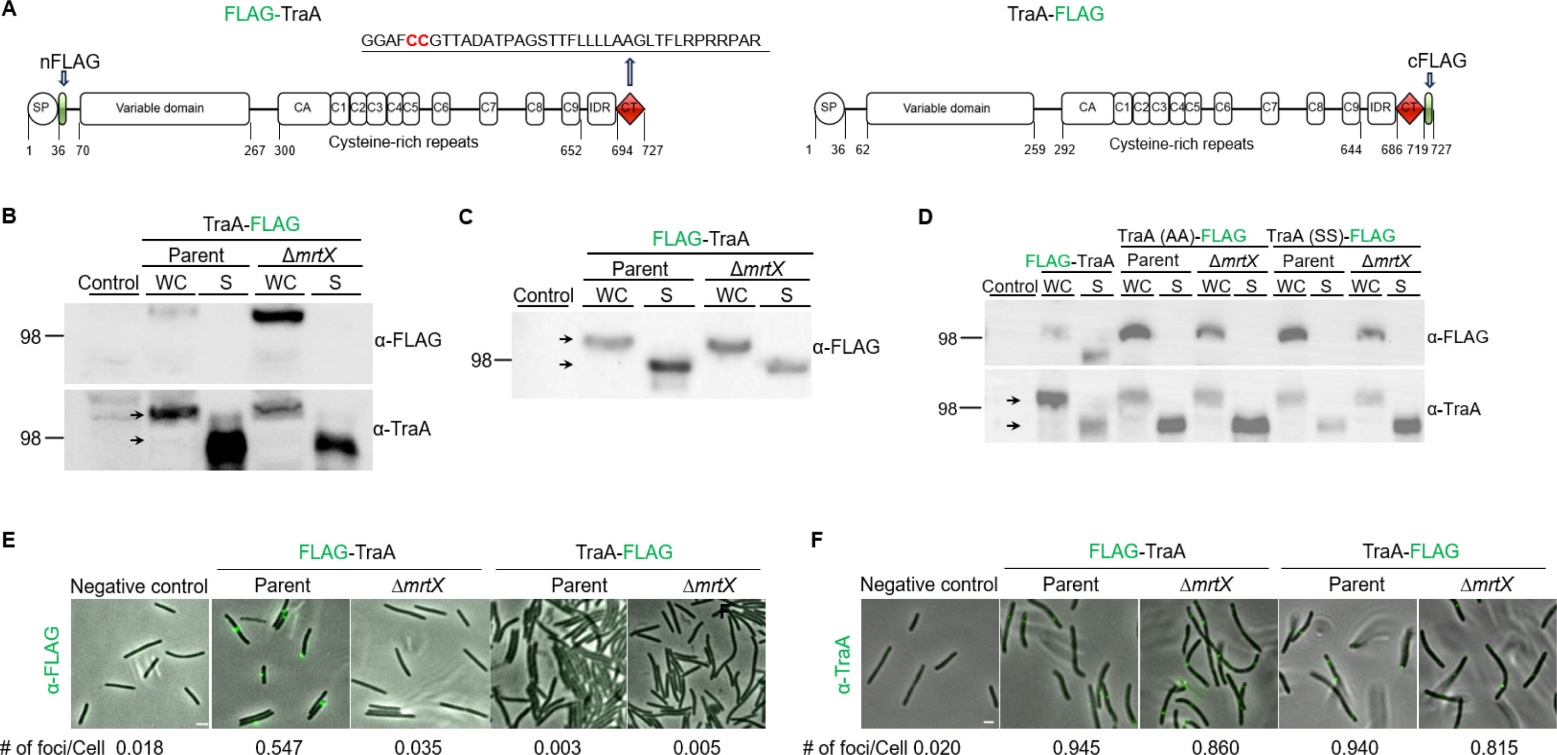
*mrtX* mutant impairs processing and cell surface localization of TraA with FLAG epitopes. (**A**) TraA domain structure and location of two different FLAG epitopes. SP, signal peptide; IDR, intrinsically disordered region; CT, MYXO-CTERM with its sequence and invariant CC residues in red. (**B**) TraA-CTERM cleaved by MrtX. Western blots of parent and Δ*mrtX* strains expressing TraA-FLAG. The whole cell lysates and secreted (S) media samples were subjected to Western analysis with indicated antibodies. The top blot was stripped and re-probed with α-TraA antibodies (bottom). Arrows mark TraA size difference (~20 kDa) between WC and S samples. Size marker, kDa. (**C**) Western blot of FLAG-TraA construct. (**D**) Western blots of parent and Δ*mrtX* strains expressing MYXO-CTERM variants TraA_CC→AA_-FLAG or TraA_CC→SS_-FLAG that block processing. Top blot stripped and re-probed with α-TraA antibodies (bottom). To compare TraA-FLAG variants processing to WT construct see panel B. (**E**) TraA live cell immunofluorescence and cell surface localization detected with α-FLAG antibodies of indicated strains. Green foci indicate TraA localized on the cell surface. Bottom, the average number of foci per cell, 300 cells counted per strain. See Table S2 for strain details. Bar, 2 µm. Representative images of three independent experiments are shown. (**F**) Detection of TraA in permeabilized Δ*mrtX* and parent strains. For foci number, 200 cells were counted per strain. Cells were incubated with α-TraA antibodies, followed by Alexa-Fluor 488-conjugated donkey anti-rabbit IgG. Negative control, no TraA epitope. Scale bar, 2 µm.

To investigate whether TraA was cleaved by MrtX, we analyzed FLAG-tagged TraA variants, in which the small FLAG epitope was engineered at the N-terminus following the signal peptide (FLAG-TraA) or at the C-terminus (TraA-FLAG) (Fig. 3A) and tested for cleavage by Western analysis. For these studies, a markerless Δ*mrtX* mutant was constructed. Strikingly, in immunoblots with α-FLAG antibodies, we detected a robust signal of TraA-FLAG in the Δ*mrtX* mutant but barely detected a signal from the parent (Fig. 3B). However, when the blot was stripped and re-probed with α-TraA antibodies, TraA was readily detected in whole cell (WC) lysates from the parent (Fig. 3B). We note that although TraA retains the C-terminal FLAG in the Δ*mrtX* mutant, and hence predicted to be 40 amino acids longer than in the parent strain, its migration on gels appeared similar between strains. In addition, TraA-FLAG was not detected in the secreted (S) media with α-FLAG antibodies from either strain, but when the blot was re-probed with α-TraA antibodies, it was readily detected in both strains (Fig. 3B), revealing that the truncated and secreted forms of TraA lack the CTERM sequence. As a control and consistent with a previous study (20), FLAG-TraA was detected in both WC lysates and S media in the Δ*mrtX* mutant and parent strains (Fig. 3C). Taken together, these results indicate that MrtX cleaves the TraA C-terminus.

### MrtX cleavage of TraA requires invariant cysteine residues

To test if the invariant cysteine within the MYXO-CTERM was required for cleavage, it was replaced with alanine or serine. In these studies, TraA was used, which actually has an adjacent cysteine (C686–C687) in its MYXO-CTERM (Fig. 3A) (20), and consequently, both residues were changed. Strikingly, there is a significant amount of TraA in WC lysates from the TraA_CC→AA_-FLAG and TraA_CC→SS_-FLAG variants, but not for the WT TraA-FLAG when probed with α-FLAG antibodies (compare Fig. 3 panel B to D). For these variants, TraA was again not detected in the secreted samples when probed with α-FLAG antibodies. However, when the blot was stripped and re-probed with α-TraA antibodies, truncated TraA variants were detected in S media independent of the myxosortase background (Fig. 3D), showing they were processed and secreted independent of MrtX. Previously, we showed that these TraA variants were impaired in cell surface localization and were nonfunctional (20). Taken together, our results show that the CC residues within the TraA MYXO-CTERM were required for proper cleavage and cell surface anchoring.

### MrtX functions as a general cell surface-sorting enzyme

Since Δ*mrtX* mutants were defective in OME, which requires TraA cell surface localization, we hypothesized that TraA was mislocalized, given that TraA was readily detected in the Δ*mrtX* mutant, though at reduced levels compared to WT (Fig. 3B). To test this, we expressed FLAG-TraA or TraA-Flag in the parent and Δ*mrtX* strains and conducted live cell immunofluorescence with α-FLAG antibodies (20). As predicted, immunofluorescence labeling showed that the average number of foci measured for FLAG-TraA in the Δ*mrtX* strain was reduced >15-fold compared to the parent (Fig. 3E). This result shows that TraA surface localization was defective and suggests that TraA was trapped intracellularly in Δ*mrtX* cells. Consistent with a previous study (20), no fluorescence was observed for TraA-FLAG on the parent (Fig. 3E), indicating the C-terminal FLAG tag was removed. Additionally, TraA-FLAG was not detected on the surface of Δ*mrtX* cells (Fig. 3E), further suggesting a secretion defect since the construct was detected by Western analysis (Fig. 3B).

To test whether TraA was trapped inside Δ*mrtX* cells, we permeabilized both the parent and Δ*mrtX* cells expressing FLAG-TraA or TraA-FLAG to allow bulky antibodies to enter cells. Fluorescence microscopy detected similar levels of FLAG-TraA or TraA-FLAG in the parent and Δ*mrtX* mutants (Fig. 3F), which contrasts with live cell immunofluorescence (Fig. 3F). Based on these observations, we conclude that the majority of TraA was unable to reach the cell surface in the Δ*mrtX* mutant.

**Fig 4:**
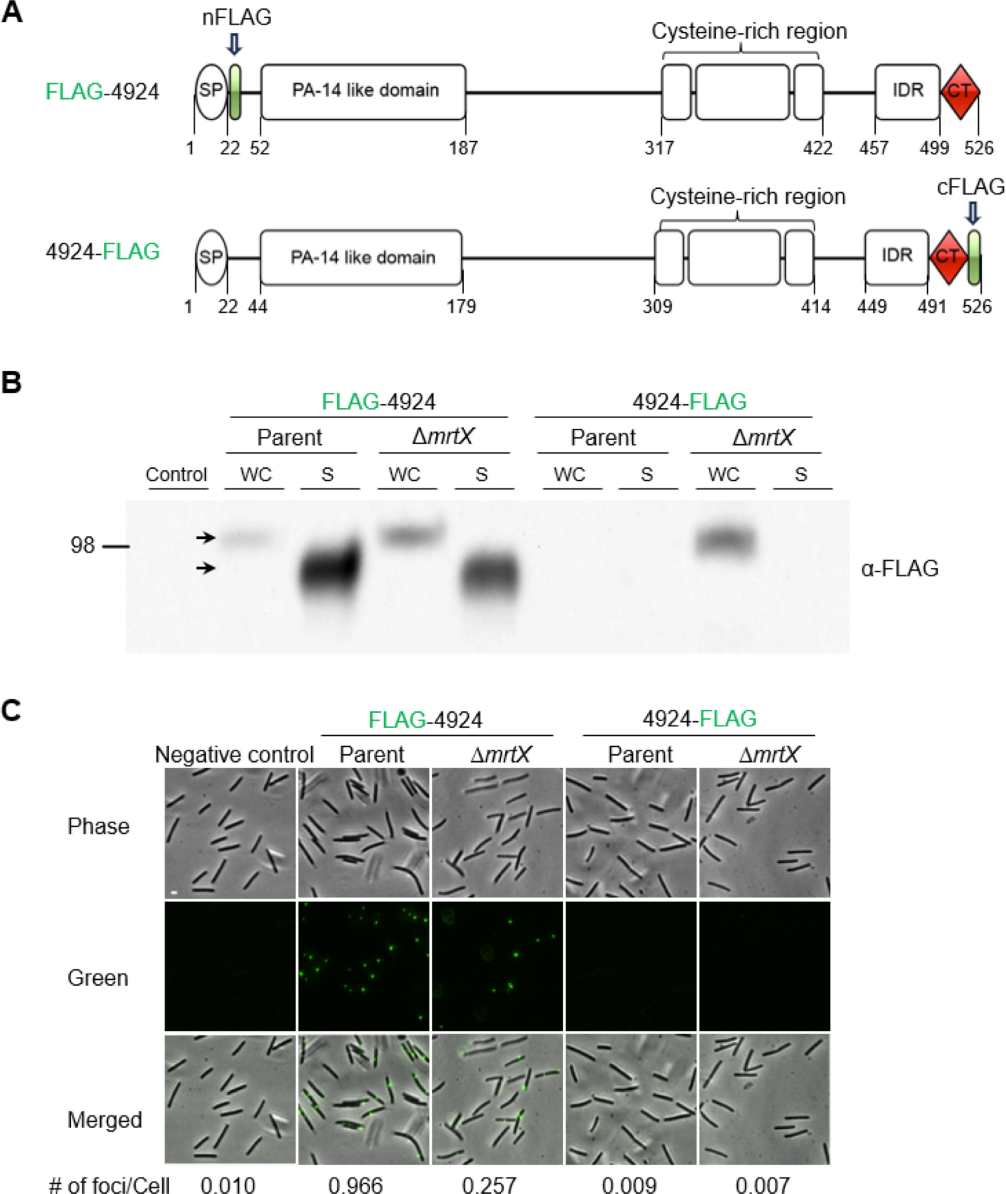
MrtX MYXO-CTERM processing from MXAN_4924. (**A**) The domain architecture of 4924 with engineered FLAG epitopes. (**B**) Western blots of parent and Δ*mrtX* strains expressing FLAG-MXAN_4924 or MXAN_4924-FLAG. Arrows indicate size difference (~20 kDa) between WC and S samples. Size marker, kDa. (**C**) Live cell immunofluorescence of surface-localized MXAN_4924 detected with FLAG antibodies. Cells treated and foci quantified as in Fig. 3E. Phase-contrast, fluorescent, and merged images are shown. Scale bar, 2 µm.

To test whether MrtX cleaved other MYXO-CTERM proteins, we used MXAN_4924 as a marker because we previously showed that it also localizes to the cell surface in a MYXO-CTERM-dependent manner (20). MXAN_4924 was similarly FLAG tagged at the N- or C-termini (Fig. 4A). Importantly, when expressed in Δ*mrtX*, MXAN_4924-FLAG was detected in WC lysates, but not in the parent strain (Fig. 4B). Similar to TraA, MXAN_4924-FLAG was not detected in either strain’s S media. As a control, FLAG-MXAN_4924 was detected in WC lysates and culture media in both strains (Fig. 4B). These results support our TraA findings and confirm that MrtX plays a role in processing MYXO-CTERM proteins. Next, we tested whether MXAN_4924 was missorted in Δ*mrtX* by live-cell immunofluorescence. Similar to TraA, FLAG-MXAN_4924 was detected with α-FLAG antibodies on the cell surface of the parent, but at reduced levels in the Δ*mrtX* mutant (Fig. 4C). As expected, MXAN_4924-FLAG was not detected in either strain (Fig. 4C). Since MXAN_4924-FLAG was readily detected in Δ*mrtX* immunoblots, we permeabilized cells before immunofluorescence labeling. With this treatment, MXAN_4924-FLAG was now detected in Δ*mrtX* but not the parent cells (Fig. S5). Taken together, these results indicate that MYXO-CTERM motifs serve as MrtX substrates that localize proteins to the cell surface.

### MrtX mutant exhibits cell envelope defects

**Fig 5:**
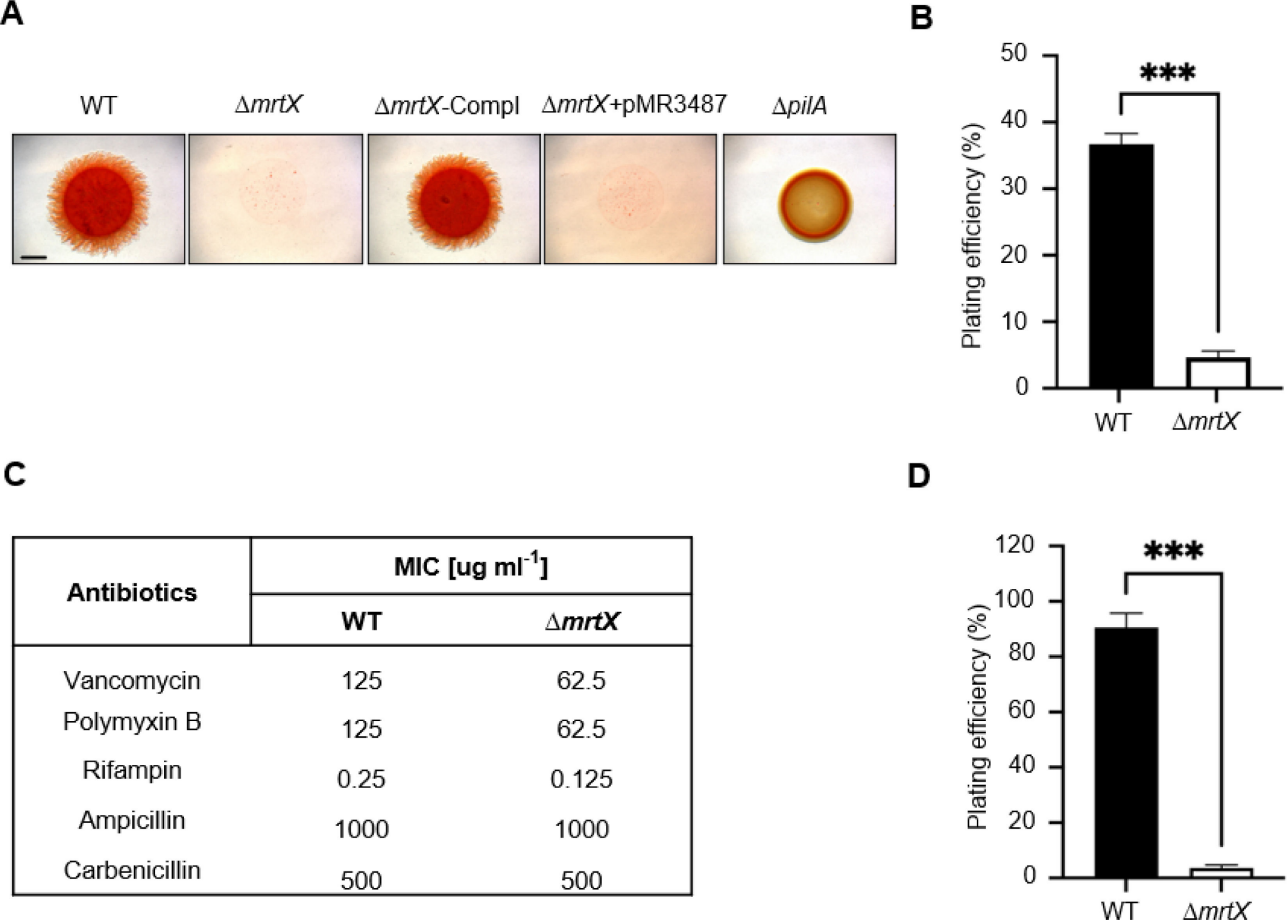
Compound sensitivity profile of Δ*mrtX* mutant. (**A**) Cells incubated on CTT 0.5% agar containing 30 µg/mL Congo red. Micrographs at 5 days. Δ*pilA* mutant serves as a negative control for EPS production and CR staining (36). WT is DK1622; see Table S2 for other strains. Scale bars, 2 mm. (**B**) Plating efficiency of WT and Δ*mrtX* strains on 20 µg/mL CR compared to CTT-only plates. Asterisks indicate a significant pairwise difference between strains by Student’s *t*-test (***P < 0.001). Error bars represent SEM; *n* = 3. (**C**) Effect of Δ*mrtX* mutations on antibiotic susceptibility. Minimum inhibitory concentration (MIC) against different antibiotics. (**D**) Plating efficiency on 25 µg/mL vancomycin compared to control plate.

To probe the global impacts of *mrtX* mutations on cell envelope integrity, we examined exopolysaccharide (EPS) production and cell permeability properties. Congo red (CR) is an anionic azo dye that binds to amyloid proteins and EPS and is routinely used in *M. xanthus* to assess EPS and fibril production (31–33). CR and its metabolites are also cytotoxic by a variety of mechanisms, including inhibiting cell wall formation and being mutagenic (34, 35). Strikingly, the Δ*mrtX* mutant exhibited a strong sensitivity to CR (Fig. 5A), while ectopic expression of the *mrtX*^+^ gene restored CR resistance (Fig. 5A). In supplemental assays to assess EPS production, we used calcofluor white (CFW) or trypan blue staining (Fig. S6) (32, 36). These assays revealed that the Δ*mrtX* mutant did not show an EPS defect. To quantify CR sensitivity, we plated cells on CR and found the plating efficiency of the Δ*mrtX* mutant was reduced approximately eightfold compared to its parent (Fig. 5B). Prior studies in *S. aureus* showed that teichoic acid mutants were highly susceptible to CR, apparently due to permeability defects caused by the loss of their cell wall anionic polymers that repel anionic compounds such as CR (35, 37).

To investigate cell permeability defects in *mrtX* mutants, we tested and found increased sensitivity to bulky or hydrophobic antibiotics, including polymyxin B and vancomycin, as well as rifampin, a lipophilic antibiotic (Fig. 5C) (38, 39). Additionally, the plating efficiency of Δ*mrtX* on vancomycin plates was ~50-fold lower compared to its parent (Fig. 5D). In contrast, for small and permeable antibiotics, such as ampicillin and carbenicillin, there was no change in MIC values.

To further test for permeability defects, we assayed sensitivity to crystal violet, a selective agent against gram-positive bacteria that lack the outer membrane permeability barrier of gram-negative bacteria, as well as outer membrane-sensitizing agents EDTA and SDS (40). These tests found that Δ*mrtX* was hypersensitive to crystal violet and EDTA, but not SDS (Fig. S7). Collectively, these findings reveal that MrtX plays an important role in maintaining cell envelope integrity, including their outer membrane permeability barrier.

### MrtX social behavior phenotypes

Several MYXO-CTERM proteins have implicated roles in cell motility and development (Table S1). We, therefore, tested for motility and developmental behaviors of the Δ*mrtX* mutant. Strains were incubated on hard agar that facilitates A-motility or on soft agar, which favors S-motility (41). As shown in Fig. S6 and 8, the Δ*mrtX* mutant had a small but significant increase in colony swarming.

Motility plays a central role in developmental aggregation, so we evaluated the ability of the Δ*mrtX* mutant to form fruiting bodies on starvation agar. In comparison to WT, the Δ*mrtX* mutant exhibited a developmental delay, while the complemented strain rescued this defect (Fig. S9). Combined, these results show that *mrtX* mutants have pleiotropic defects, including in motility and multicellular development.

### MrtX cleavage of MYXO-CTERM in a heterologous *E. coli* host

**Fig 6:**
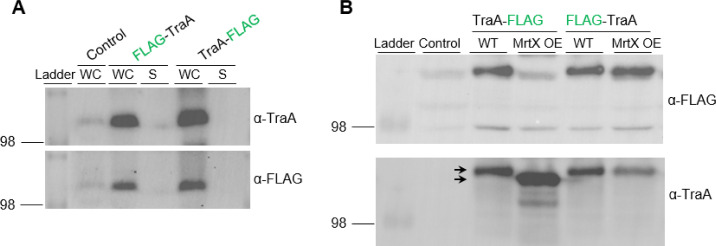
MYXO-CTERM cleaved in a heterologous *E. coli* host by MrtX. (**A**) Western blot of TraA variants in WC lysates and S media. (**B**) Detection of TraA variants in WC *E. coli* BL21(DE3) lysates with and without co-expression of *mrtX*. In panels A and B, the top blots were stripped and re-probed with indicated antibodies (bottom). Arrows indicate mature and processed TraA. Size markers, kDa.

To test whether MrtX was necessary and sufficient for MYXO-CTERM cleavage, we evaluated its activity in an *E. coli* heterologous host. In immunoblots with FLAG or TraA antibodies, TraA was readily detected in WC lysates but not S media (Fig. 6A). Additionally, neither of the TraA FLAG constructs were detected by live cell immunofluorescence (Fig. S10), indicating TraA was not secreted or cell surface localized in *E. coli*. Importantly, with α-FLAG antibodies, TraA-FLAG was readily detectable in *E. coli* in the absence of MrtX, but only weakly detected when MrtX was co-expressed (Fig. 6B). However, after the membrane was stripped and re-probed with α-TraA antibodies, TraA was detected when MrtX was co-expressed. Additionally, TraA migrated slightly faster when MrtX was co-expressed in *E. coli* (Fig. 6B). These results show that MrtX was necessary and sufficient for MYXO-CTERM cleavage in *E. coli*.

## DISCUSSION

In this work, we show that the *M. xanthus mrtX* gene encodes a sortase-like enzyme that cleaves the C-terminus of MYXO-CTERM proteins, which is required for proper protein sorting and anchoring to the cell surface. In the case of TraA, the conserved cysteine residues within MYXO-CTERM were required for MrtX cleavage. Importantly, using *E. coli* as a heterologous host, we show that MrtX is necessary and sufficient for MYXO-CTERM cleavage.

Myxosortase is a member of the glutamate intramembrane endopeptidases family. This family includes eukaryotic type II CAAX protease Rce1, a membrane-embedded endopeptidase found in yeast and the human ER involved in protein sorting and sub-cellular localization (16, 42). Family members are also widely distributed in prokaryotes but with poorly defined roles. AlphaFold 3 predicted structure indicates that MrtX has seven transmembrane helices and predictive active site glutamic acid residues, similar to MmRce1 (16). Our findings suggest that MrtX is the primary and perhaps only protease that specifically cleaves MYXO-CTERM sequences. The other two *M. xanthus* CAAX proteases have unknown functions, although some gram-positive bacteria contain multiple sortases that act on different target proteins (9).

**Fig 7:**
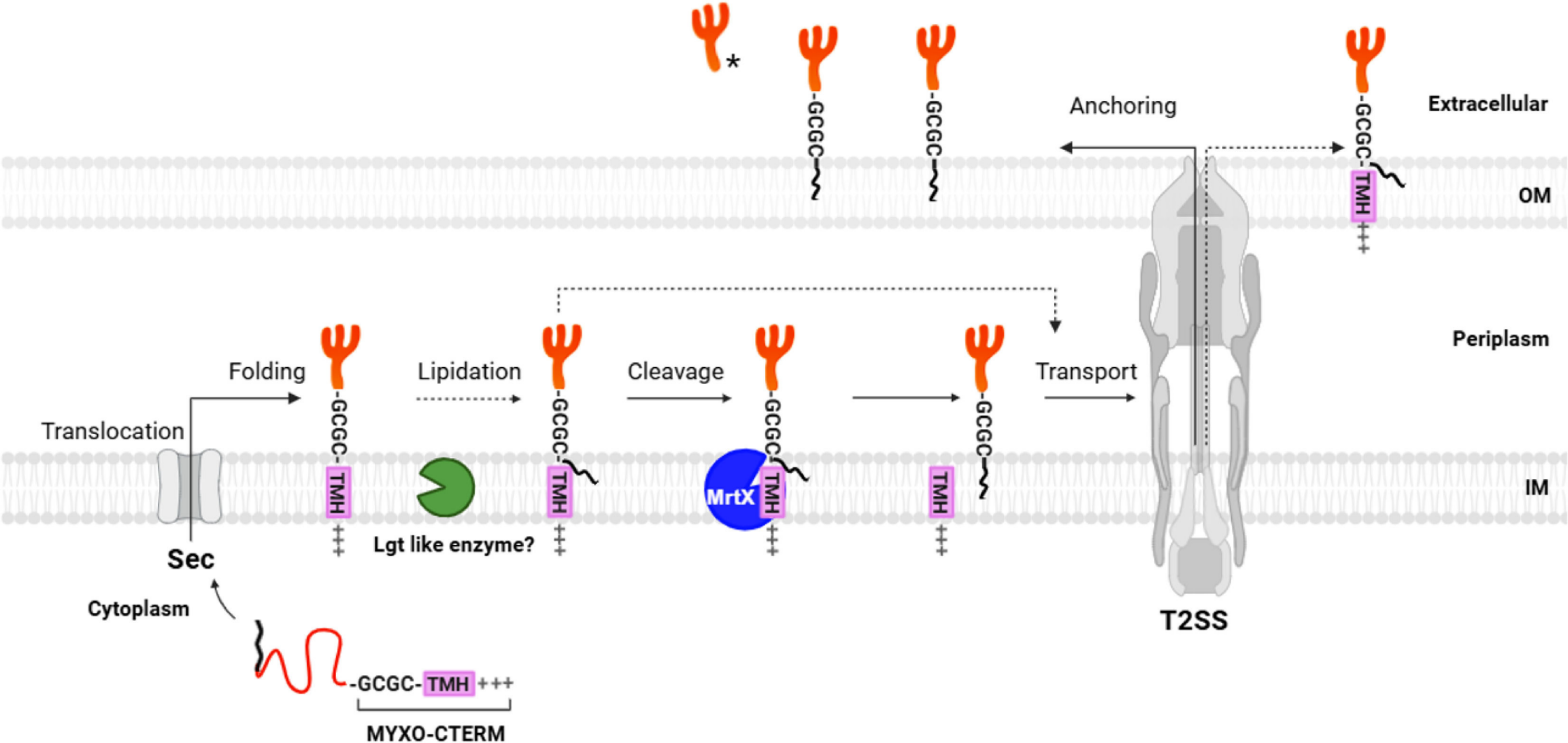
Working model of the MYXO-CTERM sorting pathway in *M. xanthus*. MYXO-CTERM protein synthesized as a precursor with N-terminal SP (black) that targets it to the Sec machinery. Following export and SP cleaved by signal peptidase, the C-terminal TMH of MYXO-CTERM anchors the protein in the IM while it folds. The MYXO-CTERM signature motif includes an invariant cysteine with a generic GCGC motif (Fig. 1A). A predicted Lgt-like acyl-transferase (green) covalently attaches a lipid moiety to the invariant cysteine. Myxosortase (MrtX, blue) then recognizes and cleaves the TMH from the MYXO-CTERM, leaving the protein anchored in the IM by its lipid tail. The folded client protein is then recognized by the T2SS and transported to the cell surface where it is anchored in the OM by its lipid moiety. In the absence of myxosortase, a small fraction of MYXO-CTERM proteins are transported to the cell surface by the T2SS, anchored in the OM by their uncleaved TMH and attached lipid. An asterisk indicates a fraction of TraA is released extracellularly by an unknown protease. Dashed lines represent speculative steps. See text for details.

We propose a model for how MYXO-CTERM proteins are processed and sorted to the cell surface (Fig. 7). First, these proteins are secreted across the IM by the Sec pathway via their N-terminal signal peptides. Following secretion, proteins are anchored in the inner membrane (IM) via their TMH within the MYXO-CTERM sequence. Analogous to other CPBP enzymes, we propose an IM-associated enzyme that covalently attaches a lipid moiety to the invariant Cys residue. Following lipidation, and analogous to the type II lipoprotein signal peptidase LspA (43), MrtX acts as an intramembrane endopeptidase that recognizes the lipidated cysteine substrate. MtrX then cleaves the distal side of the lipidated cysteine, which simultaneously releases the MYXO-CTERM peptide. The T2SS then recognizes these proteins once they fold, in a manner similar to lipoprotein and GlyGly-CTERM protein recognition, and transports them across the OM to the cell surface (15). The two-step modification, lipidation first on the Cys side chain and then myxosortase-catalyzed cleavage of the polypeptide chain, differs from the one-step transpeptidation done by sortase.

MYXO-CTERM-containing proteins were identified bioinformatically in all examined genera that belong to the phylum Myxococcota (16, 20). Similar to the GlyGly-CTERM proteins from *V. cholerae*, processed by rhombosortase, MYXO-CTERM proteins rely on the T2SS for transport across the OM for cell surface localization (20). These transport pathways are similar to how the T2SS transports particular lipoproteins to the cell surface, such as pullulanase from *Klebsiella oxytoca* (44).

In an atypical configuration, MXAN_5598 contains an internal MYXO-CTERM motif (Table S1), which may have resulted from the fusion of two genes. The N-terminal region contains a predicted large adhesin region followed by a MYXO-CTERM motif, while the C-terminal region encodes an OM β-barrel and OmpA cell wall-binding domains. This domain configuration is similar to the combined architecture of the TraA and TraB proteins, whose ORFs overlap (21). As C-terminal sorting tags are functional even if they reside in the middle of a protein (45, 46), we similarly propose that the N-terminal portion of MXAN_5598 is processed and anchored to the cell surface. Curiously, MXAN_5598 lies in the middle of a large cell division gene cluster, adjacent to *ftsA* and *ftsZ*, suggesting a link between cell attachment and cell cycle control.

Myxosortase functions in an analogous manner as sortase, archaeosortase, and rhombosortase found in other bacteria and archaea phyla. All of these enzymes process a C-terminal tripartite architecture of their substrates, which consists of a short recognition motif, followed by a hydrophobic region and a stretch of positively charged cytoplasmic residues. However, these families of protein-sorting endopeptidases differ in their active site residues, the sorting signals they recognize, and posttranslational modifications.

Rhombosortase (RssP) is a member of the rhomboid family of intramembrane serine proteases that possibly attaches the substrate protein to a phosphatidylethanolamine on the newly generated C-terminus via transamidation (15). Archaeosortase (ArtA) mediates PGF-CTERM processing of the S-layer glycoprotein in *H. volcanii* (14). Like SrtA, ArtA has an active site cysteine and a similar catalytic triad (47). ArtA cleaves the C-terminus for the subsequent attachment of a prenylation reaction (13). However, despite extensive characterization, it remains unclear the exact processing site or the lipid moiety that attaches to this glycoprotein (14). Like myxosortase, Rce1 and MmRce1 are members of the CAAX protease family. Rce1 (Ras and a-factor-converting enzyme) is an endopeptidase that only cleaves the CAAX proteins when the Cys residue is farnesylated or geranylgeranylated by a separate enzyme (48). MmRce1, the archaeal homolog of Rce1, is also an endopeptidase that specifically cleaves the CAAX box after the cysteine residue undergoes prenylation in *M. maripaludis* (27). Since proteins containing CAAX motifs are often lipidated on the cysteine residue by a separate enzyme before protease cleavage (49), we similarly hypothesize that MYXO-CTERM-containing proteins are lipidated prior to and without dependence on the action of myxosortase. Future studies need to elucidate the mechanism of MYXO-CTERM modification and determine whether myxosortase protease activity depends on lipidation by other enzymes.

TraA and MXAN_4924 are defective in cell surface localization in the Δ*mrtX* mutant, but, interestingly, the defect is not absolute. There are at least two plausible explanations. First, secondary cell envelope protease(s) could cleave in the MYXO-CTERM region and, in particular, their adjacent intrinsically disordered region (Fig. 3A and 4A in reference 20). Alternatively, the T2SS extracts noncleaved MYXO-CTERM proteins from the IM and transports them to the cell surface (Fig. 7). Here, their transmembrane domain and presumed posttranslational modification anchor them in the OM.

TraA and MXAN_4924 are also secreted extracellularly, similar to the GlyGly-CTERM protein VesB. VesB is an extracellular serine protease of *V. cholerae*, which cleaves and activates cholera toxin (50). Cleavage of VesB by an alternative rhomboid protease GlpG, instead of RssP, results in an inactive form that is released into the extracellular space following translocation by the T2SS (51). In *S. aureus*, cell surface-attached sortase substrate SPA is shed from the cell envelope and released into the culture supernatant by murein hydrolases (52). Surface-localized TraA and MXAN_4924 could similarly undergo processing and release by a cell surface or extracellular protease. Alternatively, aberrant cleavage could occur in the periplasm, which consequently results in their secretion by the T2SS (20).

Our *in silico* work identified MrtX as the candidate myxosortase we experimentally confirmed here. However, as predicted by our models and comparative genomics, a cognate myxosortase acyl-transferase remains unidentified. For this reason, we hypothesized that the putative acyl-transferase is a broadly conserved enzyme across phyla. To test this, we assayed MrtX activity in an *E. coli* heterologous host, which contains no MYXO-CTERM proteins or MrtX orthologs. Strikingly, MrtX processed the TraA MYXO-CTERM motif in *E. coli*. This result supports our hypothesis where a leading acyl-transferase candidate is Lgt, a widely conserved and essential protein that lipidates proteins at their invariant cysteine residue (2), which LspA subsequently cleaves. Although there are intriguing parallels between these pathways, there are substrate differences, including the absence of a defined lipobox and an inverted topology of MYXO-CTERM proteins in the cytoplasmic membrane. Two other explanations we cannot exclude, but we think are unlikely for processing in *E. coli* are (i) the MYXO-CTERM does not require posttranslational modification for cleavage by MrtX, or (ii) MrtX has dual functions and posttranslationally modifies its substrate before cleavage. Future work seeks to elucidate MYXO-CTERM posttranslational processing in *E. coli* and *M. xanthus*.

Intriguingly, in addition to MrtX function in the TraA-mediated OME pathway, our results revealed that MrtX functions in other pathways including motility, development, and cell envelope permeability. These pleiotropic phenotypes are consistent with myxosortase acting on 35 diverse classes of MYXO-CTERM proteins. For example, the sensitivity of MrtX mutants to CR, crystal violet, and bulky/hydrophobic antibiotics suggests that some of MYXO-CTERM proteins, which are not properly processed, play a role in cell envelope integrity. Moreover, because the Δ*mrtX* mutation does not abolish cell surface localization and function of TraA and MXAN_4924, other MXYO-CTERM proteins likely retain partial localization and functions, hence reducing the severity of MrtX phenotypes.

## MATERIALS AND METHODS

### Bacterial strains and growth conditions

Table S2 lists bacterial strains and plasmids used in this study. *M. xanthus* strains were grown in the dark at 33°C with shaking in CTT media (1% [wt/vol] casitone, 10 mM Tris-HCl pH 7.6, 1 mM KH_2_PO_4_, and 8 mM MgSO_4_). *E. coli* cultures were grown in LB medium at 37°C. For solid media plates, agar was added at 1.5% or 0.5% (wt/vol). Antibiotics were added as needed: 50 µg/mL kanamycin (Km) for *M. xanthus* and *E. coli*, 15 µg/mL oxytetracycline (oTc) for *M. xanthus*, and 10 µg/mL tetracycline (Tc) for *E. coli*. TPM buffer (CTT without casitone) was used to wash cells.

### Plasmid and strain construction

Primers used in this study are listed in Table S3. To create insertion mutations, approximately 500 bp of the gene of interest was PCR amplified, and the amplicon was ligated into the pCR-XL-TOPO vector (Life Technologies) and electroporated into *E. coli* DH5α. Plasmids were verified by PCR, restriction analysis, and sequencing. Verified constructs were electroporated into *M. xanthus* and recombined into the chromosome by homologous recombination.

In-frame deletions of *mrtX* were constructed by a two-step homologous recombination method. Briefly, ~500 bp upstream and downstream of *mrtX* was PCR amplified and cloned into a linearized vector pBJ114 (53) (digested with EcoRI and HindIII) via Gibson assembly (New England Biolabs). The resulting plasmid, pBJ114-*mrtX*, containing the *mrtX* deletion cassette and the Km^R^-*galK* selection-counter-selection cassette, was electroporated into *M. xanthus* cells. Recombinants were selected by Km^R^ and then counter-selected for the loss of *galK* on 3% galactose plates (54). Deletions were confirmed by PCR with flanking primers and phenotypic analysis.

To create an *mrtX* complementation strain, the *mrtX* ORF was PCR amplified and cloned into pMR3487 (55), downstream of the IPTG-inducible promoter using the XbaI and KpnI restriction sites. Verified constructs were electroporated into *M. xanthus*, and transformants were selected by oTc^R^ and confirmed by phenotypic analysis.

To generate MXAN_4924-FLAG fusions, a FLAG epitope tag was engineered at the C-terminus of MXAN_4924 by PCR, and the amplicon was ligated into both pMR3487 and pSWU19 vectors. Constructs were verified and electroporated into *M. xanthus* as described.

### Stimulation assay

This assay was performed as described (56). Briefly, *M. xanthus* was grown overnight to the mid-log phase. Nonmotile nonstimulatable donor strains were mixed with nonmotile stimulatable recipient strains and placed onto 1/2 CTT (0.5% casitone) agar plates supplemented with 2 mM CaCl_2_. After incubation at 33°C, the edges of colonies were imaged with a Nikon E800 phase contrast microscope with a 10× objective lens.

### Western blotting

Western blotting was performed as described (20) with a few modifications. Briefly, exponentially growing *M. xanthus* cells were harvested, washed twice in TPM buffer, and then resuspended in 1× SDS buffer to a density of 4.5 × 10^9^ cells per mL. Spent culture media were precipitated in ice-cold trichloroacetic acid and incubated on ice for 30 min. Pellets were washed twice in ice-cold acetone. Pellets were air dried and resuspended in 1× SDS buffer before heating at 95°C for 10 min.

For *E. coli*, cells were grown overnight in LB at 37°C, diluted to an OD_600_ of 0.02, followed by 6 h growth at 30°C with or without 1 mM IPTG. WC pellets were taken and resuspended in sample buffer equivalent to 10 OD_600_ before heating at 95°C for 10 min. Spent culture media were prepared as described above.

To generate rabbit polyclonal anti-TraA sera, two TraA peptides, TADSCGPGCVKCPGERPY and SAKTMECERGRRKPGTEP, were synthesized as antigens. Pre-immune sera from rabbits were screened by Western analyses against *M. xanthus* WC lysates to select rabbits with minimal background cross-reactivity prior to immunization (Thermo Scientific Pierce Protein Biology). Primary affinity purified anti-TraA serum was used at 1:800 dilution and polyclonal anti-FLAG antibody (Sigma-Aldrich) at 1:1,500 dilution. For detection, horseradish peroxidase (HRP)-conjugated goat anti-rabbit secondary antibody (Pierce) was used at 1:15,000 dilution. Protein transfer was done with Trans-Blot Turbo (BioRad). Blots were developed with HRP substrate (Millipore) and visualized using a BioRad ChemiDoc MP image analyzer.

### Microscopy and immunofluorescence

These assays were generally performed as described (18). Briefly, exponentially growing cells were harvested, washed twice with TPM, and blocked with 2% BSA for 30 min. Primary α-FLAG antibody (1:600 dilution) was added for a 1.5 h incubation, washed, and resuspended in 300 µL blocking solution (TPM with 2% BSA) containing 1 µL of secondary antibody (Alexa Fluor 488-conjugated donkey anti-rabbit IgG; Jackson ImmunoResearch) for 1.5 h incubation in the dark. Finally, cells were washed four times in TPM. All incubations were done at room temperature with gentle end-to-end rocking.

#### Immunofluorescence on fixed and permeabilized cells

Briefly, cells were fixed with 1.6% paraformaldehyde and 0.025% glutaraldehyde for 10 min. Following fixation, cells were washed in PBS buffer (137 mM NaCl, 2.7 mM KCl, 10 mM Na_2_HPO_4_, and 1.8 mM KH_2_PO_4_, pH 7.4), and permeabilized with 0.2 µg/mL lysozyme in GTE buffer (50 mM glucose, 10 mM EDTA, and 20 mM Tris, pH 7.5) for 4 min on a poly-L-lysine-coated diagnostic slide. Cells were then blocked in PBS with 2% BSA for 20 min and probed with primary α-FLAG (1:300) or α-TraA serum (1:3,000) for 1 h at room temperature. Alexa Fluor 488-conjugated donkey anti-rabbit IgG (1:300) was used as a secondary antibody for a 1 h incubation. Slow antifade reagent (Thermo Fisher Scientific) was added to the slide before immunofluorescence imaging. Fluorescent microscopy was done on glass slides with a Nikon E800 microscope equipped with a 100× or 60× oil objective lens and FITC or Texas Red filter sets.

### Congo red sensitivity assay

*M. xanthus* was grown in CTT with shaking at 33°C to the mid-log phase. Cells were washed and resuspended in TPM buffer to a density of 3 × 10^8^ cells per mL. Twenty microliter aliquots of cell suspension were plated onto CTT 0.5% agar plates containing 30 µg mL^−1^ CR (57). Images were captured after 5 days of incubation at 33°C using an Olympus SZX10 stereomicroscope coupled to a digital imaging system.

To determine plating efficiency, cells were harvested, washed, and subjected to 10-fold serial dilutions in TPM buffer. A volume of 10 µL of each dilution was spotted onto CTT 1.5% agar plates containing 20 µg mL^−1^ CR. The plates were air-dried and incubated at 33°C for 5 days. Plating efficiency was calculated as the ratio of viable colonies to the number of initially plated cells.

### EPS assay

Exponentially growing *M. xanthus* cultures were harvested and resuspended in TPM to a calculated density of 3 × 10^8^ cells mL^−1^. Twenty-microliter aliquots were spotted on 0.5% agar plates supplemented with 0.5% CTT and 20 or 50 µg mL^−1^ of trypan blue or CFW, respectively (32). Plates were incubated at 33°C and imaged at 5 days.

### Plating efficiency

Exponentially growing cultures of *M. xanthus* were washed and 10-fold serially diluted in 96-well plates to 10^−5^. Dilutions were spotted as 4 µL drops on CTT agar, with or without 1 mg/L crystal violet, 0.156 mM EDTA, or 0.0005% SDS. The plates were incubated at 33°C for 5 days. Plating efficiency was calculated as described above.

### Antibiotic susceptibility

MIC assays were done in triplicate in 96-well plates with indicated antibiotics (58, 59). Antibiotic concentrations were initiated at 500 µg mL^−1^ (ampicillin, 1,000 µg mL^−1^) and serially diluted across each row in twofold steps until 0.06 µg mL^−1^ was reached. Each well had 3 × 10^5^ CFU/mL. After 3 days of incubation at 33°C, growth was visually assessed (turbidity) and compared to no-antibiotic wells. Control wells, with no cells, were tested for compound precipitation. MICs were scored as the lowest antibiotic concentration that inhibited visible growth/turbidity. To assess the plating efficiency, cells were 10-fold serially diluted and spotted on CTT 1.5% agar plates containing 25 µg mL^−1^ vancomycin. Plating efficiency was calculated as described above.

### Motility assays

Exponentially growing cultures of *M. xanthus* were washed and resuspended in TPM buffer to a density of 7.5 × 10^8^ cells per mL. Cells were then spotted on CTT plates with 1.5% or 0.3% agar to assess A- and S-motility, respectively. At indicated times, colony images were taken.

### Fruiting body formation

*M. xanthus* cells in the mid-log phase were pelleted by centrifugation and resuspended in TPM buffer to a density of 1.5 × 10^9^ cells per mL. Twenty microliter aliquots of cell suspension were spotted onto TPM 1.5% agar plates and incubated at 33°C. Fruiting body development was imaged at indicated times.

## SUPPLEMENTAL MATERIAL

Supplemental Material mbio.04067-24-s0001.docx Supplemental figures and tables.

ASM does not own the copyrights to Supplemental Material that may be linked to, or accessed through, an article. The authors have granted ASM a non-exclusive, world-wide license to publish the Supplemental Material files. Please contact the corresponding author directly for reuse.
