# Supplementary material for: Myxosortase: an intramembrane protease that sorts MYXO-CTERM proteins to the cell surface
Source: mBio. 2025 Mar 12;16(4):e04067-24. doi: 10.1128/mbio.04067-24 (PMC11980579; doi:10.1128/mbio.04067-24)
Supplement: Supplemental Material — Supplemental figures and tables. [file mbio.04067-24-s0001.docx]

Supplementary Material

Myxosortase: An intramembrane protease that sorts MYXO-CTERM proteins to the cell surface

Tingting Guo^1^, Daniel H. Haft^2^, and Daniel Wall^1#^

^1^Department of Molecular Biology, University of Wyoming, Laramie, WY 82071, USA

^2^National Center for Biotechnology Information, National Library of Medicine, National Institutes of Health, Bethesda, MD 20892, USA

#Corresponding author: [dwall2@uwyo.edu](mailto:dwall2@uwyo.edu)


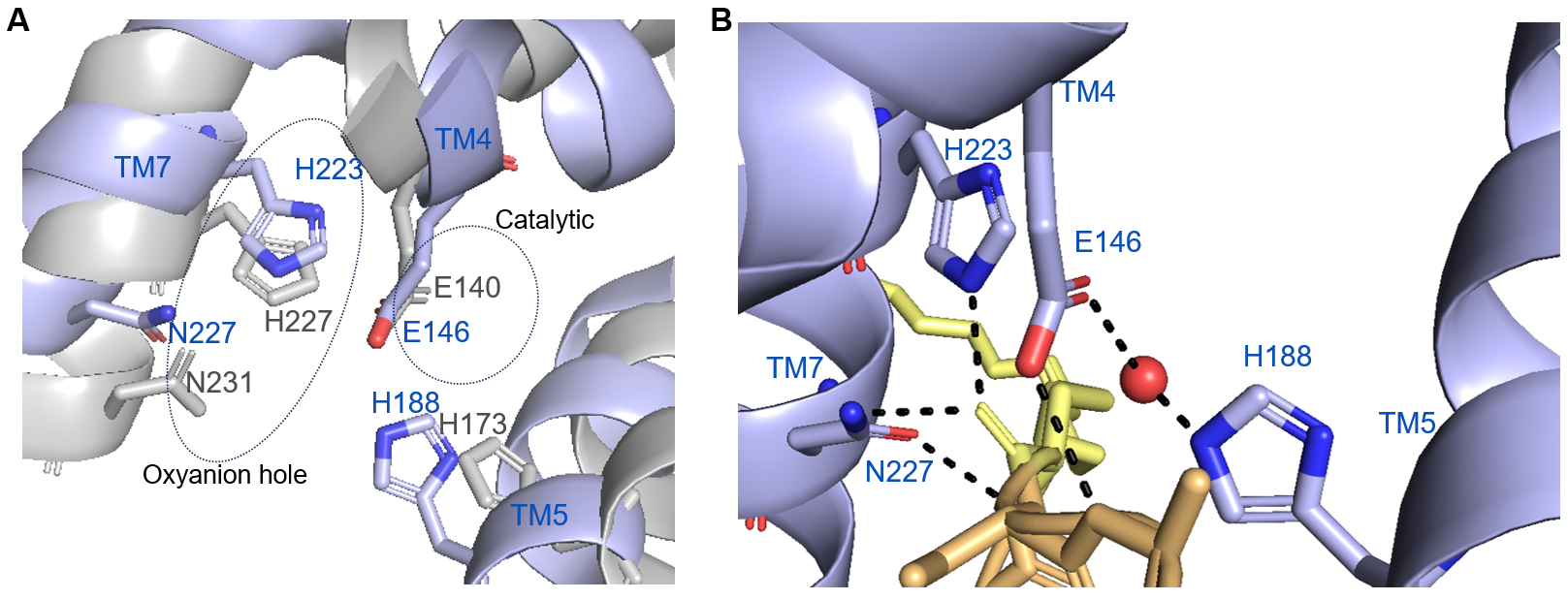
**Figure S1.** Predicted structure of the CPBP domain active site in MrtX. (A) Active sites superimpositions of MmRce1 (grey) and myxosortase (blue). Images generated in PYMOL [1]. The side chains of the four invariant CPBP domain residues shown as sticks. (B) Interactions of the MXYO-CTERM substrate (gold) with the conserved catalytic residues (E140 and H173). Palmitoyl lipid yellow, hydrogen bonds as dashed lines and water as a red sphere.


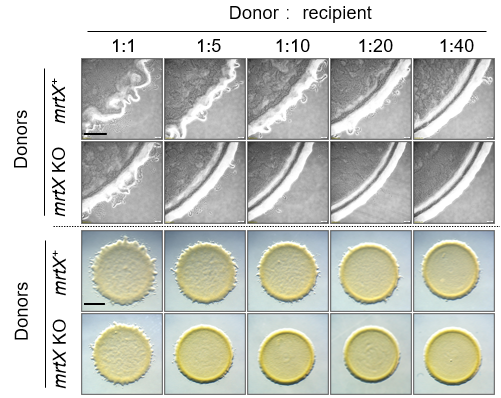


**Figure S2**. Sensitivity of stimulation assays. *mrtX*^+^ or *mrtX* knockout donor strains were mixed with a *mrtX*^+^ recipient strain at different ratios. Images at 24 h (top) or 3 days (bottom); scale bars, 200 μm and 2 mm, respectively. See Table S2 for strain details.

^
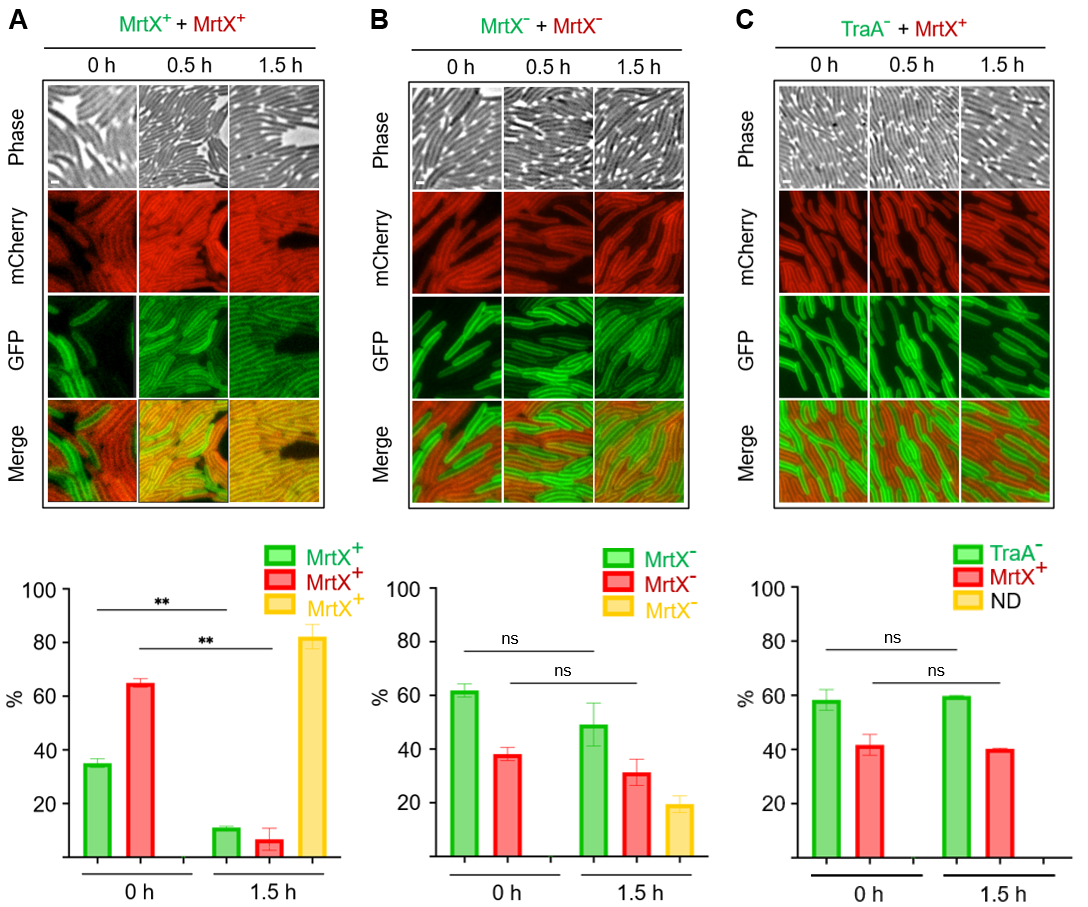
^

**Figure S3.** MrtX phenotype assessed by TraA mediated OME between cells harboring transferable SS_OM_-GFP or SS_OM_-mCherry reporters. Supplementary figure to Fig. 2B with single and merged channels. Samples were quantified by their fluorescence color at two time points. Positive control (A), *mrtX* mutants (B) and negative control (C). Scale bar = 1 µm; ND, not detected. Asterisks indicate significant pairwise differences between strains according to Student’s t-test (**P < 0.05; ns, no significant difference).


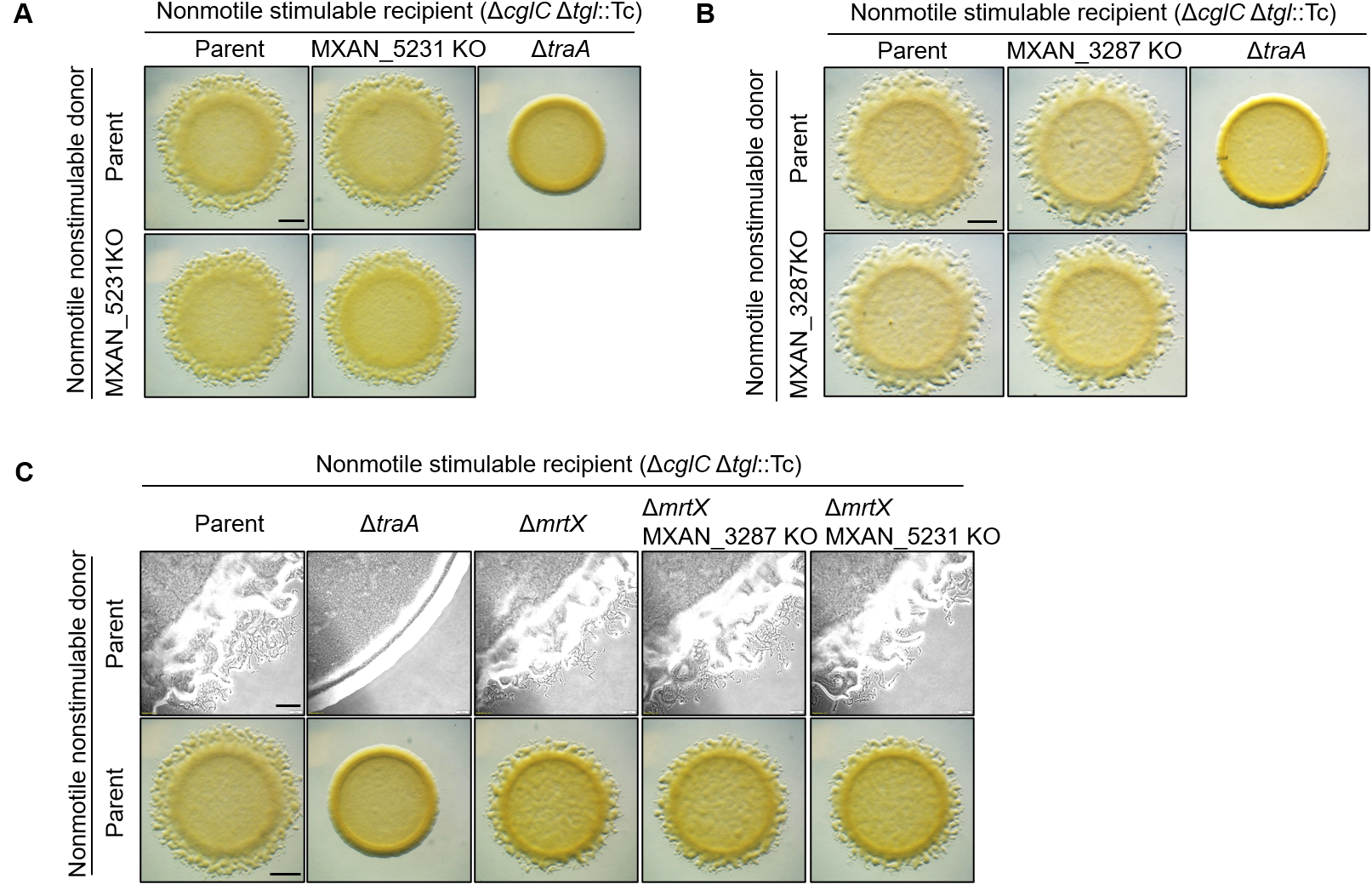


**Figure S4.** CAAX proteases MXAN_5231 and MXAN_3287 are not involved in TraA mediated OME. Stimulation activity of MXAN_5231 (A) and MXAN_3287 (B) knockouts in donor, recipient, or both strains. Positive (parent donor and recipient) and negative (recipient lacking *traA*) controls shown. Scale bars, 2 mm. (C) Knockout MXAN_5231 or MXAN_3287 in the Δ*mrtX* background phenocopied Δ*mrtX* mutant. Scale bars, 200 μm (top) and 2 mm (bottom).


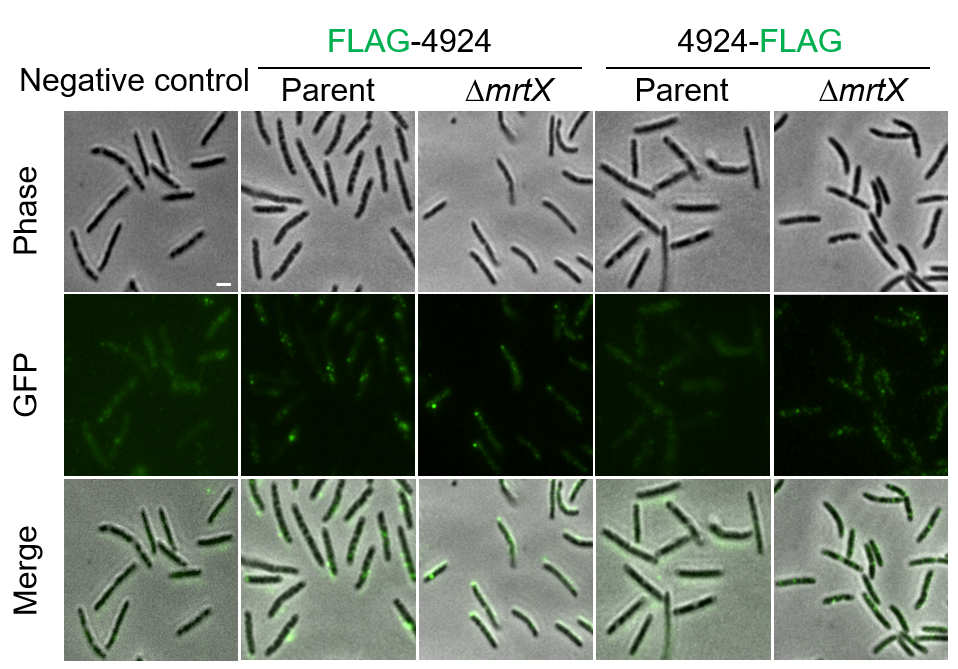


**Figure S5.** Localization of MXAN_4924 in permeabilized Δ*mrtX* and parent strains. Green foci indicate relative abundance of the FLAG-MXAN_4924 reporter. MXAN_4924-FLAG was absence in MrtX^+^ cells but present in Δ*mrtX* mutants. Permeabilized cells incubated with α-FLAG antibodies, followed by Alexa-Fluor 488-conjugated donkey α-rabbit IgG. Negative control, no FLAG epitope. Phase-contrast, immunofluorescence and merged images shown. Scale bar, 1 μm.


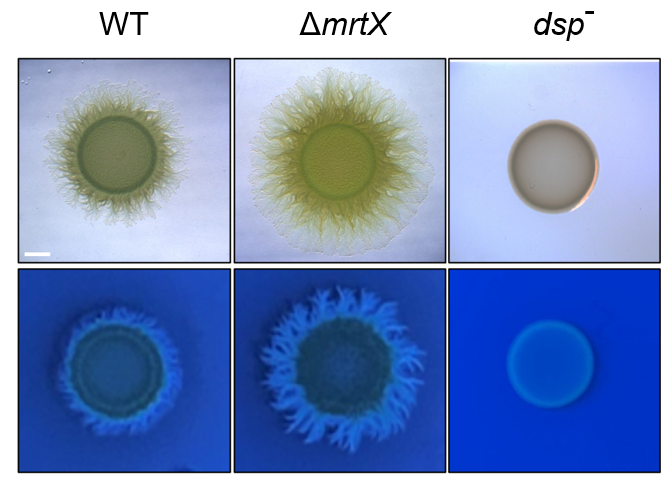


**Figure S6.** Exopolysaccharide production of WT and Δ*mrtX* strains. A *dsp* mutant, defective in EPS synthesis, used as a negative control [2]. Micrographs after three-day incubation on 20 µg/ml trypan blue (top) or 50 µg/ml CFW (bottom). Scale bar, 2 mm.


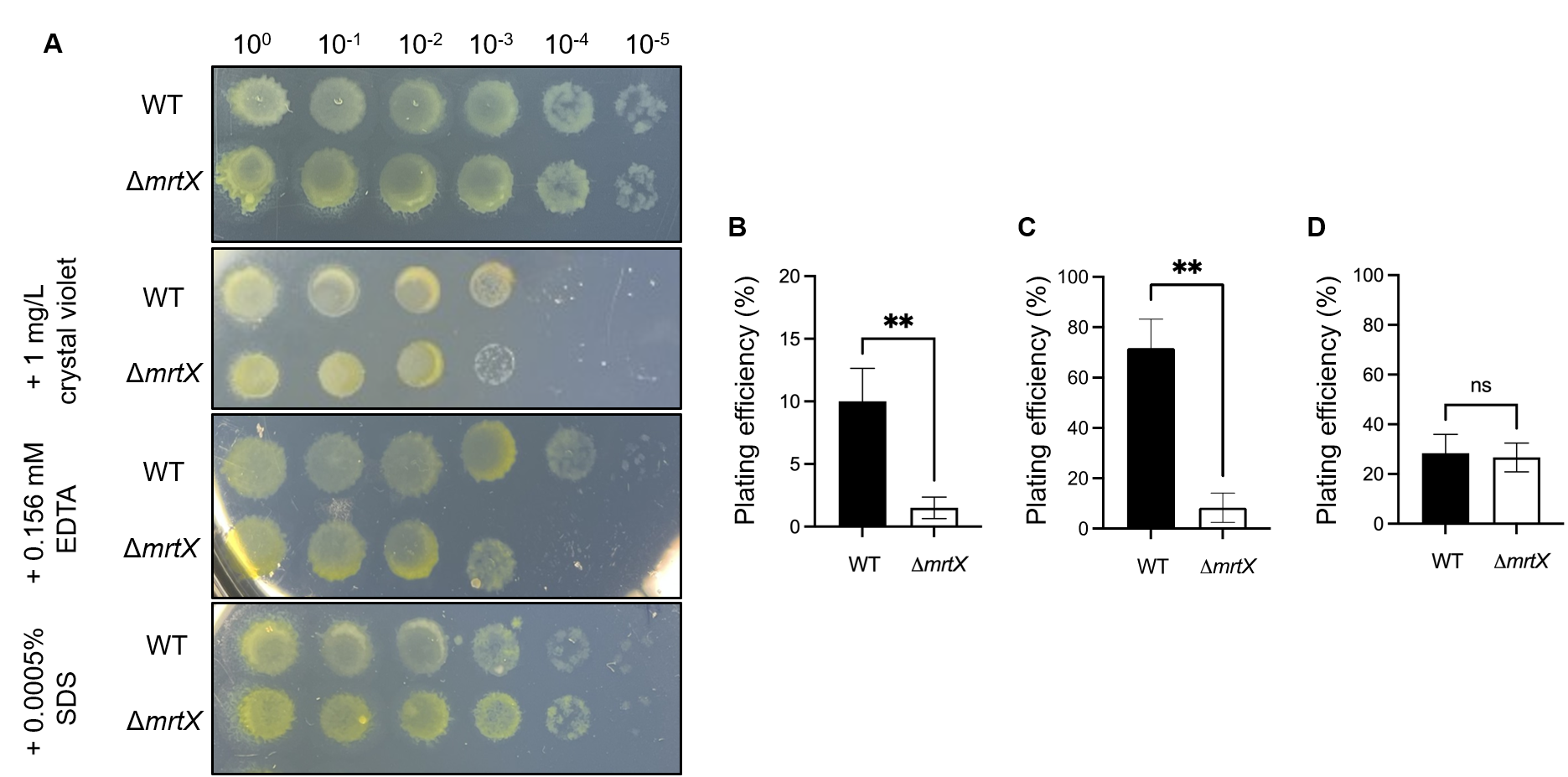


**Figure S7.** Plating efficiency assays. (A) WT (DK1622) and Δ*mrtX* strains were serially diluted and spotted on CTT plates without and with crystal violet, EDTA or SDS. Plates incubated at 33°C for 5 days. Calculated plating efficiency in the presence of 1 mg/L crystal violet (B), 0.156 mM EDTA (C) and 0.0005% SDS (D). Asterisks indicate significant pairwise difference between strains according to Student’s t test (**P < 0.05; ns, no significant difference).


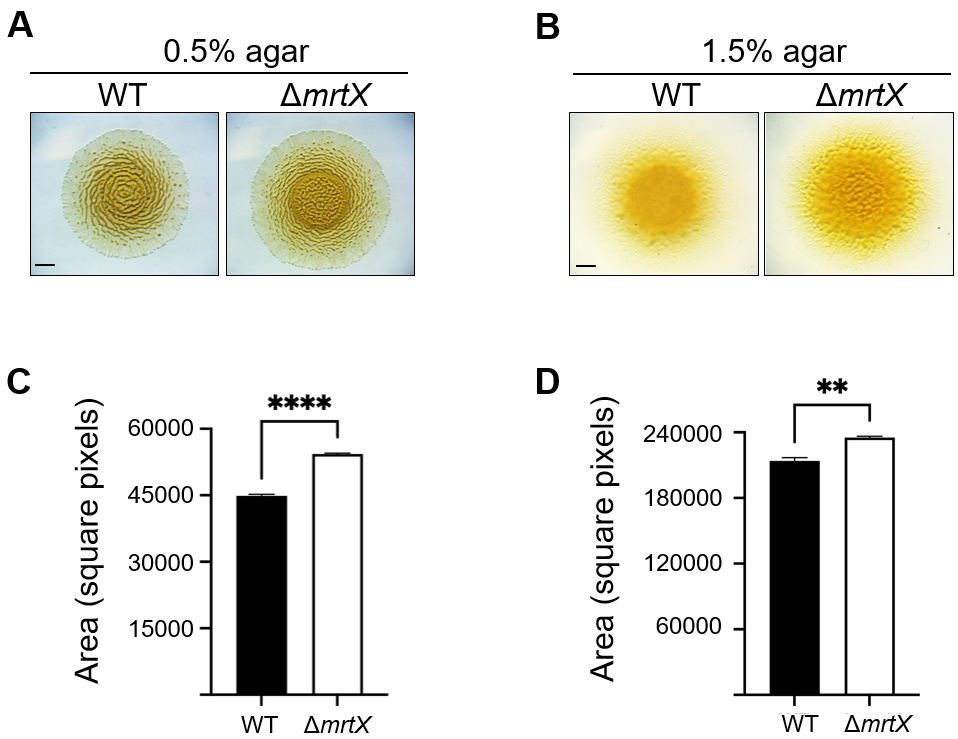


**Figure S8.** *mrtX* mutation increases motility on soft agar (A) and hard agar (B) in WT background (DK1622). Micrographs at 72 h. Scale bars, 2 mm. SD from three biological replicates shown. Asterisks indicate significant pairwise difference between strains according to Student’s t test (**P < 0.05; ****P < 0.0001).


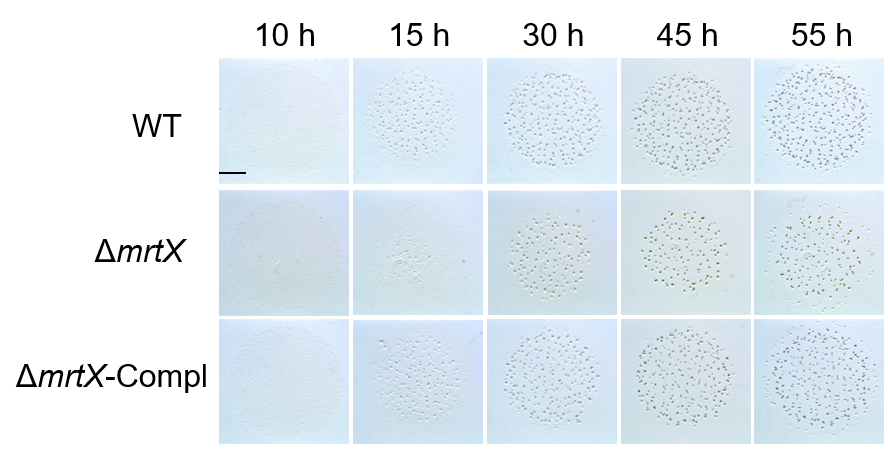


**Figure S9.** Delayed fruiting body development by *mrtX* mutant. Micrographs imaged at indicated time. Scale bar, 1 mm.


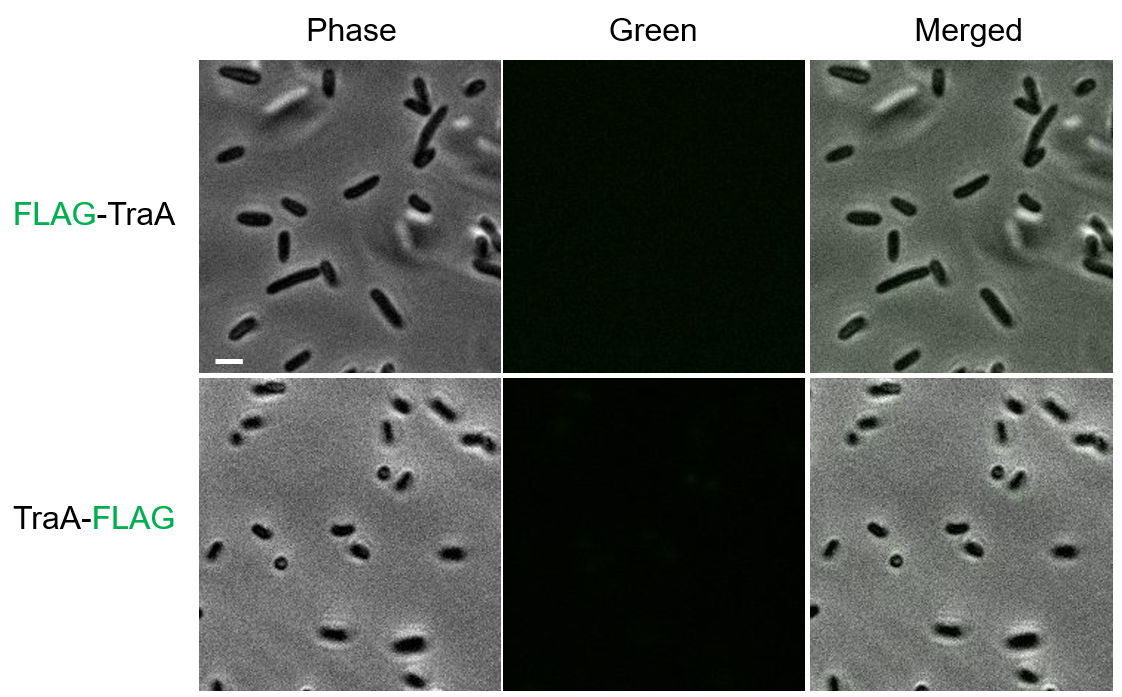


**Figure S10.** TraA does not localize on the *E. coli* cell surface. Cells treated with α-FLAG antibodies, followed by Alexa-Fluor 488-conjugated donkey α-rabbit IgG. Single-channel and merged images shown. See Fig. 6 for TraA expression levels. Scale bar, 2 μm.

**Table S1** MYXO-CTERM proteins in *M. xanthus* DK1622 genome.
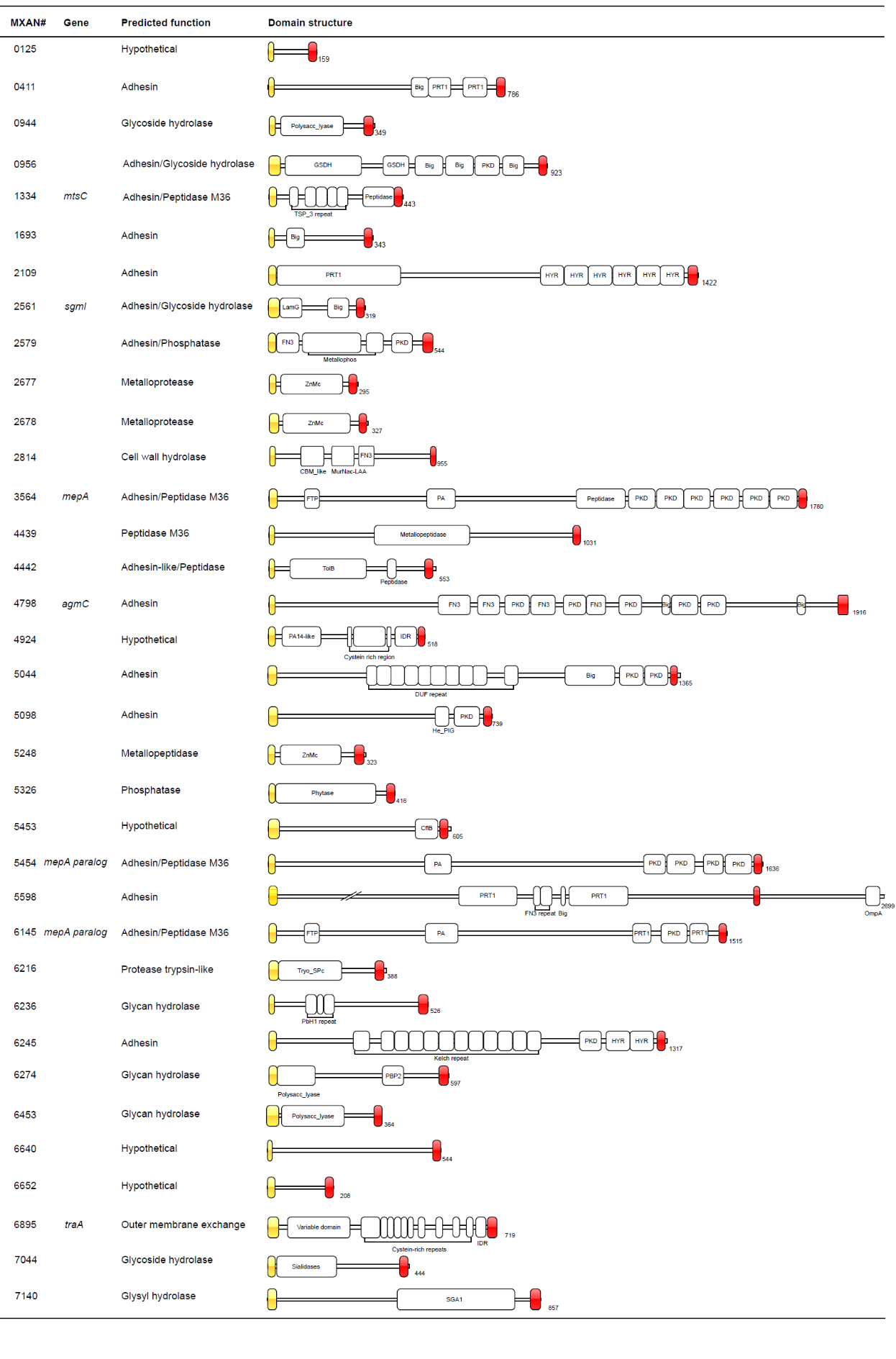


Signal peptide, yellow; MYXO-CTERM, red. Big, bacterial Ig-like domain; PRT1, internal repeat; GSDH, glucose/sorbosone dehydrogenases; PKD, polycystic kidney disease; TSP_3 repeat, thrombospondin type 3 repeat; HYR, hyalin repeat; LamG, laminin G-like; FN3, fibronectin type 3; ZnMc, zinc-dependent metalloprotease; CBM_like, carbohydrate-binding modules like; MurNac-LAA, N-acetylmuramoyl-L-alanine amidase; FTP, fungalysin/thermolysin propeptide; PA, protease associated; DUF, domain of unknown function; He_PIG, putative Ig domain; CflB, clumping factor B; Tryo_SPc, trypsin-like serine protease; PbH1 repeat, parallel beta-helix repeats; PBP2, penicillin-binding protein 2; SGA1, glucan 1,4-alpha-glucosidase.

**Table S2** Strains and plasmids used in this study.

| **Plasmids** | **Relevant features** | | **Source** |
| --- | --- | --- | --- |
| pMR3487 | IPTG-inducible promoter, Tc^R^ |  | [3] |
| pPC51 | P*_pilA_*-RBSsyn-Flag-*traA* in pSWU19, Km^R^ |  | [4] |
| pDP421 | P*_pilA_*-RBSsyn-*traA*-Flag in pSWU19, Km^R^ |  | [4] |
| pGS114 | P*_pilA_*-RBSsyn-Flag-*MXAN_4924* in pSWU19, Km^R^ |  | [4] |
| pXW6 | P*_pilA_*-*SS_OM_*-*mCherry* in pKSAT, Sm^R^ |  | [5] |
| pPC43 | P*_pilA_*-*SS_OM_*-*gfp* in pKSAT, Sm^R^ |  | [6] |
| pPC58 | pMR3487-(ATG)-*traAB*, Tc^R^ |  | [7] |
| pDP21 | P*_pilA_*-*traAB* in pSWU19 (Mx8 *attP*), Km^R^ |  | [8] |
| pTG2901 | Δ*mrtX* cassette in pBJ114, *galK* Km^R^ |  | This study |
| pTG2902 | pMR3487-*mrtX*, Tc^R^ |  | This study |
| pTG2903 | pMR3487-*MXAN_4924*-FLAG, Tc^R^ |  | This study |
| pTG2904 | *mrtX* fragment in pCR-XL-TOPO, Zeo^R^, Km^R^ |  | This study |
| pTG2905 | *MXAN_3287* fragment in pCR-XL-TOPO, Zeo^R^, Km^R^ |  | This study |
| pTG2906 | *MXAN_5231* fragment in pCR-XL-TOPO, Zeo^R^, Km^R^ |  | This study |
| pTG2907 | P*_pilA_*-RBSsyn-*traA*-Flag, (CC to AA) in pSWU19, Km^R^ |  | This study |
| pTG2908 | P*_pilA_*-RBSsyn-*traA*-Flag, (CC to SS) in pSWU19, Km^R^ |  | This study |
| **Strains** | **Relevant features** | **Experimental use** | **Source** |
| DH5α | *E. coli* cloning strain | Cloning | Lab collection |
| BL21(DE3) | *E. coli* protein expression strain | Fig. 6 | Lab collection |
| DK8601 | *aglB1* (*aglQ1*) Δ*pilA*::Tc, nonmotile, Tc^R^ | Fig. 5 | [9] |
| DK6204 | DK1622 Δ*mglBA* (markerless), nonmotile | Fig. 2, 4, S2, S4 and S6 | [10] |
| DW1466 | DK1622 Δ*tgl*::Tc Δ*cglC* (markerless), nonmotile, Tc^R^ | Fig. 2, S2 and S4 | [8] |
| DW2220 | DW1466 ∆*traA* (markerless), Tc^R^ | Fig. 2 and S4 | [11] |
| DK1622 | Wild-type *M. xanthus*, motile | Fig. 5, S7, S8 and S9 | [12] |
| DW2302 | DK6204 Δ*traA* | Fig. 3 and S5 | Lab collection |
| DK3468 | *dsp-1680*, fibril deficient, A^+^, EPS¯ mutant | Fig. S7 | [2] |
| DW2270 | DW10410 ∆*traAB* (markerless) |  | [6] |
| DW2303 | DW2270 (pPC43) | Fig. 2 and S3 | Lab collection |
| DW2290 | DK1622 pPC58, Tc^R^ |  | [13] |
| DW2901 | DK6204 (pTG2904), Zeo^R^, Km^R^ | Fig. 2 and S2 | This study |
| DW2902 | DW1466 (pTG2904), Zeo^R^, Km^R^ | Fig. 2 | This study |
| DW2903 | DK6204 (pTG2905), Zeo^R^, Km^R^ | Fig. S4 | This study |
| DW2904 | DW1466 (pTG2905), Zeo^R^, Km^R^ | Fig. S4 | This study |
| DW2905 | DK6204 (pTG2906), Zeo^R^, Km^R^ | Fig. S4 | This study |
| DW2906 | DW1466 (pTG2906), Zeo^R^, Km^R^ | Fig. S4 | This study |
| DW2907 | DW1466 Δ*mrtX* | Fig. S4 | This study |
| DW2925 | DW1466 Δ*mrtX* (pTG2905) | Fig. S4 | This study |
| DW2926 | DW1466 Δ*mrtX* (pTG2906) | Fig. S4 | This study |
| DW2908 | DK6204 Δ*pilA* |  | This study |
| DW2909 | DK6204 Δ*mrtX* Δ*pilA* |  | This study |
| DW2910 | DW2908 (pPC51) | Fig. 3 and S5 | This study |
| DW2911 | DK6204 Δ*mrtX* Δ*pilA* (pPC51) | Fig. 3 and S5 | This study |
| DW2912 | DK6204 Δ*pilA (*pDP421) | Fig. 3 and S5 | This study |
| DW2913 | DK6204 Δ*mrtX* Δ*pilA* (pDP421) | Fig. 3 and S5 | This study |
| DW2914 | DW1466 (pGS114) | Fig. 4 and S6 | This study |
| DW2915 | DW1466 Δ*mrtX* (pGS114) | Fig. 4 and S6 | This study |
| DW2916 | DK6204 (pTG2903) | Fig. 4 and S6 | This study |
| DW2917 | DK6204 Δ*mrtX* (pTG2903) | Fig. 4 and S6 | This study |
| DW2918 | DK1622 Δ*mrtX* | Fig. 5, S7, S8 and S9 | This study |
| DW2919 | DK1622 Δ*mrtX* (pTG2902) | Fig. 5, and S9 | This study |
| DW2920 | DK1622 Δ*mrtX* (pMR3487) | Fig. 5 | This study |
| DW2921 | DW2290 pXW6 | Fig. 2 and S3 | This study |
| DW2922 | DW2270 (pPC43) (pDP21) | Fig. 2 and S3 | This study |
| DW2923 | DW2270 (pPC43) (pDP21) (pTG2904) | Fig. 2 and S3 | This study |
| DW2924 | DK1622 (pXW6) (pTG2904) | Fig. 2 and S3 | This study |
| DW2925 | DK6204 Δ*pilA* (pTG2907) | Fig. 3 | This study |
| DW2926 | DK6204 Δ*mrtX* Δ*pilA* (pTG2907) | Fig. 3 | This study |
| DW2927 | DK6204 Δ*pilA* (pTG2908) | Fig. 3 | This study |
| DW2928 | DK6204 Δ*mrtX* Δ*pilA* (pTG2908) | Fig. 3 | This study |
| TG2900 | BL21(DE3) (pPC51) | Fig. 6 and S10 | This study |
| TG2901 | BL21(DE3) (pPC51) (pTG2902) | Fig. 6 | This study |
| TG2902 | BL21(DE3) (pDP421) | Fig. 6 and S10 | This study |
| TG2903 | BL21(DE3) (pDP421) (pTG2902) | Fig. 6 | This study |

**Table S3.** Primers used in this study.

| **Primer name** | **Sequence (5’→3’)*** |
| --- | --- |
| *mrtX* KO-F | GAAGCGGTGGGATTGTGG |
| *mrtX* KO-R | AGCGCCGTCAGCCAGAAG |
| MXAN_3287 KO-F | CGAACCCAGCCTTCGG |
| MXAN_3287 KO-R | CACGGCGGAGATGACC |
| MXAN_5231 KO-F | TGCTCGCCTGGGGACTG |
| MXAN_5231 KO-R | CGAACTCCTCGAAGCCTG |
| Δ*mrtX*-EcoRI-UF | GTAAAACGACGGCCAGTGAATTCTGCTCAACACGCTCCCG |
| Δ*mrtX-*UR | GAGAAACCGCACATAGAGCAATCCCACCGCTTCCTG |
| Δ*mrtX* Dn-F | AGGAAGCGGTGGGATTGCTCTATGTGCGGTTTCTC |
| Δ*mrtX*-HindIII-Dn-R | TATGACCATGATTACGCCAAGCTTCGGTGGTGCTCTTGACC |
| Δ*mrtX*-verification-F | TTCGGGACGGCGACAGCCTTGA |
| Δ*mrtX*-verification-R | TGAGCCTCGGAAGCAGCAGTGGA |
| *mrtX*-compl-UF-XbaI | GCGCTCTAGACATGACCCAGGCCGTGACG |
| *mrtX*-compl-DR-KpnI | CCGGGGTACCCAACGCAATAAGCCATTCTCG |
| pSWU19-MXAN_4924-XbaI-F | GGCTCTAGAGGAAACCAAGAATAGAAATAGAAAGGAGAATTAATGC  GATTGCTCCGCATC |
| pSWU19-MXAN_4924-FLAG-HindIII-R | GCCAAGCTTCTACTTGTCGTCGTCGTC |
| pMR3487-MXAN_4924-XbaI-F | CTAGTCTAGAATGCGATTGCTCCGCATC |
| pMR3487-MXAN_4924-FLAG-KpnI-R | ATGGGTACCCTACTTGTCGTCGTCGTCCTTGTAGTCTCGCCGACCGCG  TCTCAC |
| TraA-XbaI-F | GGTCTAGAGGAAACCAAG |
| TraA-before-CC-R | GAAGGCGCCGCCTTGAGG |
| TraA-CCtoAA-F | CACCGAGCCTCAAGGCGGCGCCTTCGCCGCCGGCACCACCGCCGACG |
| TraA-CCtoSS-F | CACCGAGCCTCAAGGCGGCGCCTTCAGCAGCGGCACCACCGCCGACG |
| TraA-Flag-HindIII-R | TTGTAAAACGACGCCAAGCTTTCACTTGTCGTCATCGTCTTTGTAGTCT  CGAGCAGGGCGCCGG |

Restriction sites underlined.

**References:**

1. L. Schrodinger. 2015. The PyMOL Molecular Graphics System.

2. Dana JR, Shimkets LJ. 1993. Regulation of cohesion-dependent cell interactions in *Myxococcus xanthus.* J Bacteriol 175:3636-3647. https://doi.org/10.1128/jb.175.11.3636-3647.1993.

3. Iniesta AA, García-Heras F, Abellón-Ruiz J, Gallego-García A, Elías-Arnanz M. 2012. Two systems for conditional gene expression in *Myxococcus xanthus* inducible by isopropyl-β-D-thiogalactopyranoside or vanillate*.* J Bacteriol 194:5875-85. https://doi.org/10.1128/jb.01110-12.

4. Sah GP, Cao P, Wall D. 2020. MYXO-CTERM sorting tag directs proteins to the cell surface via the type II secretion system*.* Mol Microbiol 113:1038-1051. https://doi.org/10.1111/mmi.14473.

5. Wei X, Pathak DT, Wall D. 2011. Heterologous protein transfer within structured myxobacteria biofilms*.* Mol Microbiol 81:315-326. https://doi.org/10.1111/j.1365-2958.2011.07710.x.

6. Cao P, Wei X, Awal RP, Müller R, Wall D. 2019. A highly polymorphic receptor governs many distinct self-recognition types within the *Myxococcales* order*.* mBio 10:e02751-18. https://doi.org/10.1128/mBio.02751-18.

7. Balagam R, Cao P, Sah GP, Zhang Z, Subedi K, Wall D, Igoshin OA. 2021. Emergent myxobacterial behaviors arise from reversal suppression induced by kin contacts*.* mSystems 6:e00720-21. https://doi.org/10.1128/mSystems.00720-21.

8. Pathak DT, Wei X, Bucuvalas A, Haft DH, Gerloff DL, Wall D. 2012. Cell contact-dependent outer membrane exchange in myxobacteria: genetic determinants and mechanism. PLoS Genet 8:e1002626. https://doi.org/10.1371/journal.pgen.1002626.

9. Wall D, Kaiser D. 1998. Alignment enhances the cell-to-cell transfer of pilus phenotype. Proc Natl Acad Sci U S A 195:3054-3058. https://doi.org/10.1073/pnas.95.6.3054.

10. Hartzell P, Kaiser D. 1991. Upstream gene of the *mgl* operon controls the level of MglA protein in *Myxococcus xanthus.* J Bacteriol 173:7625-7635. https://doi.org/10.1128/jb.173.23.7625-7635.1991.

11. Cao P, Wall D. 2017. Self-identity reprogrammed by a single residue switch in a cell surface. Proc Natl Acad Sci U S A 114:3732–3737. https://doi.org/10.1073/pnas.1700315114.

12. Dey A, Vassallo CN, Conklin AC, Pathak DT, Troselj V, Wall D. 2016. Sibling rivalry in *Myxococcus xanthus* is mediated by kin recognition and a polyploid prophage. J Bacteriol 198:994-1004. https://doi.org/10.1128/jb.00964-15.

13. Vassallo CN, Sah GP, Weltzer ML, Wall D. 2021. Modular lipoprotein toxins transferred by outer membrane exchange target discrete cell entry pathways*.* mBio 12:10.1128/mbio. 02388-21. https://doi.org/10.1128/mbio.02388-21.
